# Microglia dynamics in retinitis pigmentosa model: formation of fundus whitening and autofluorescence as an indicator of activity of retinal degeneration

**DOI:** 10.1038/s41598-020-71626-2

**Published:** 2020-09-07

**Authors:** Kenichi Makabe, Sunao Sugita, Michiko Mandai, Yoko Futatsugi, Masayo Takahashi

**Affiliations:** 1 Laboratory for Retinal Regeneration, Center for Biosystems Dynamics Research, RIKEN, 2-2-3 Minatojima-minamimachi, Chuo-ku, Kobe, 650-0047 Japan; 2 Department of Ophthalmology, Kobe Kaisei Hospital, 3-11-15 Shinoharakitamachi, Nada-ku, Kobe, 657-0068 Japan

**Keywords:** Cell biology, Diseases, Pathogenesis

## Abstract

In patients with retinitis pigmentosa (RP), color fundus photography and fundus autofluorescence (FAF) have been used to estimate the disease progression. To understand the origin and the diagnostic interpretation of the fundus color and FAF, we performed in vivo imaging of fundus color and FAF together with histological analyses of the retinal degeneration process using the RP model mice, *rd10*. FAF partly represented the accumulation of microglia in the photoreceptor outer segments. Fundus whitening suggested the presence of apoptotic cells, which spatiotemporally preceded increase in FAF. We observed two patterns of FAF localization, arcuate and diffuse, each indicating different pattern of apoptosis, wavy and diffuse, respectively. Diffuse pattern of apoptosis was suppressed in dark-raised *rd10* mice, in which outer nuclear layer (ONL) loss was significantly suppressed. The occupancy of FAF correlated with the thinning rate of the ONL. Fractalkine, a microglia chemotactic factor, was detected in apoptotic photoreceptors, suggesting chemokine-induced recruitment of microglia into the ONL, which paralleled with accelerated ONL loss and increased FAF occupancy. Thus, we propose that the degree of photoreceptor apoptosis and the rate of ONL thinning in RP patients might be read from the fundus color and the FAF.

## Introduction

Retinitis pigmentosa (RP) is a hereditary progressive retinal degeneration that can cause blindness. The patient is estimated over one million worldwide. The disease is characterized by primary degeneration of rod photoreceptor cells due to a gene mutation related to the function or the structure of the photoreceptors and the retinal pigment epithelium (RPE)^1^. Over 70 genes are now reported as causal genes of the disease (RetNet: https://sph.uth.edu/retnet/).

By short wave fundus autofluorescence (FAF) imaging, a hyperfluorescent ring is often observed in the eye of RP patients, which coincides with the border of the ellipsoid-zone-deficient site detected by optical coherence tomography (OCT)^2^. The hyperfluorescent ring constricts over time^3^ and the radius correlates with the amplitude in pattern electroretinogram (ERG)^4^. Therefore, FAF imaging is expected to provide a key parameter to estimate the degree of photoreceptor degeneration in RP patients in clinical practice. However, what this hyperfluorescence represents is still unknown. Although it was pointed out that autofluorescent RPE cells may contribute to hyperautofluorescent ring observed in human RP^3^, the involvement of another autofluorescent cell, microglia, has not been investigated. In a previous study of other retinal degenerated model mice, the appearance of fundus autofluorescent spots originated from microglia is reported^5^. Therefore, it is possible that in RP, microglia may be another contributor to the autofluorescent ring.

Microglia is a parenchymal tissue macrophage in the central nervous system (CNS) including retina. CD68^+^ microglia/macrophage collected from the subretinal fluid of patients with retinal detachment exhibited autofluorescence^6^. Phagocytic microglia also showed autofluorescence by in vivo imaging in animal models of retinal degeneration^5,7^. Retinal microglia normally reside in the inner layer (INL) of the retina, but they are known to infiltrate into the outer nuclear layer (ONL) in RP animal models^8^ and RP patients^9,10^. In the retina of retinal degeneration 10 (*rd10*) mice, microglia infiltrate into the ONL to phagocytose apoptotic photoreceptors. It is known that the number of microglia in the ONL correlates with the number of apoptotic photoreceptors in the ONL^10^. If the behavior of microglia could be monitored by in vivo imaging, it may be a good indicator for apoptosis of the photoreceptors. It is also reported that chemokines such as fractalkine/CX3CL1^11^ and MCP-1/CCL2^12^ are expressed in apoptotic photoreceptors and recruit microglia to the degenerating ONL in a light damaged retinal degeneration model. However, it is unknown whether these chemokines play a part to attract microglia in the retinas of RP patients.

We therefore hypothesized that in RP pathology, microglia that receive signals from degenerating photoreceptors infiltrate into the ONL to phagocytose photoreceptors and cause FAF, thus can be a biomarker for real-time photoreceptor degeneration. In order to verify the hypothesis, we performed three experiments using *rd10* mice: (1) in vivo FAF imaging during the retinal degeneration, (2) histopathological examination of retinal specimen at the same time points with in vivo FAF imaging to investigate the related pathological event such as apoptosis of the photoreceptors and infiltration of microglia, and (3) analyses of the expression levels and the localization of microglial chemotactic factors, fractalkine and MCP-1. The *rd10* strain shares a common missense mutation with human RP patents in the *cGMP phosphodiesterase β subunit* (*PDE6β*) gene and exhibit retinal degeneration similar to human RP^13^. Since it is known that protection from light is effective in *rd10* mice to delay the progress of the disease^13^, we also bred *rd10* mice in dark to examine the parameters change in vivo, which are presumably involved in the delay of the disease.

## Results

### Whitish retina and increased autofluorescence was observed in retinitis pigmentosa model: *rd10* mice

**Figure 1:**
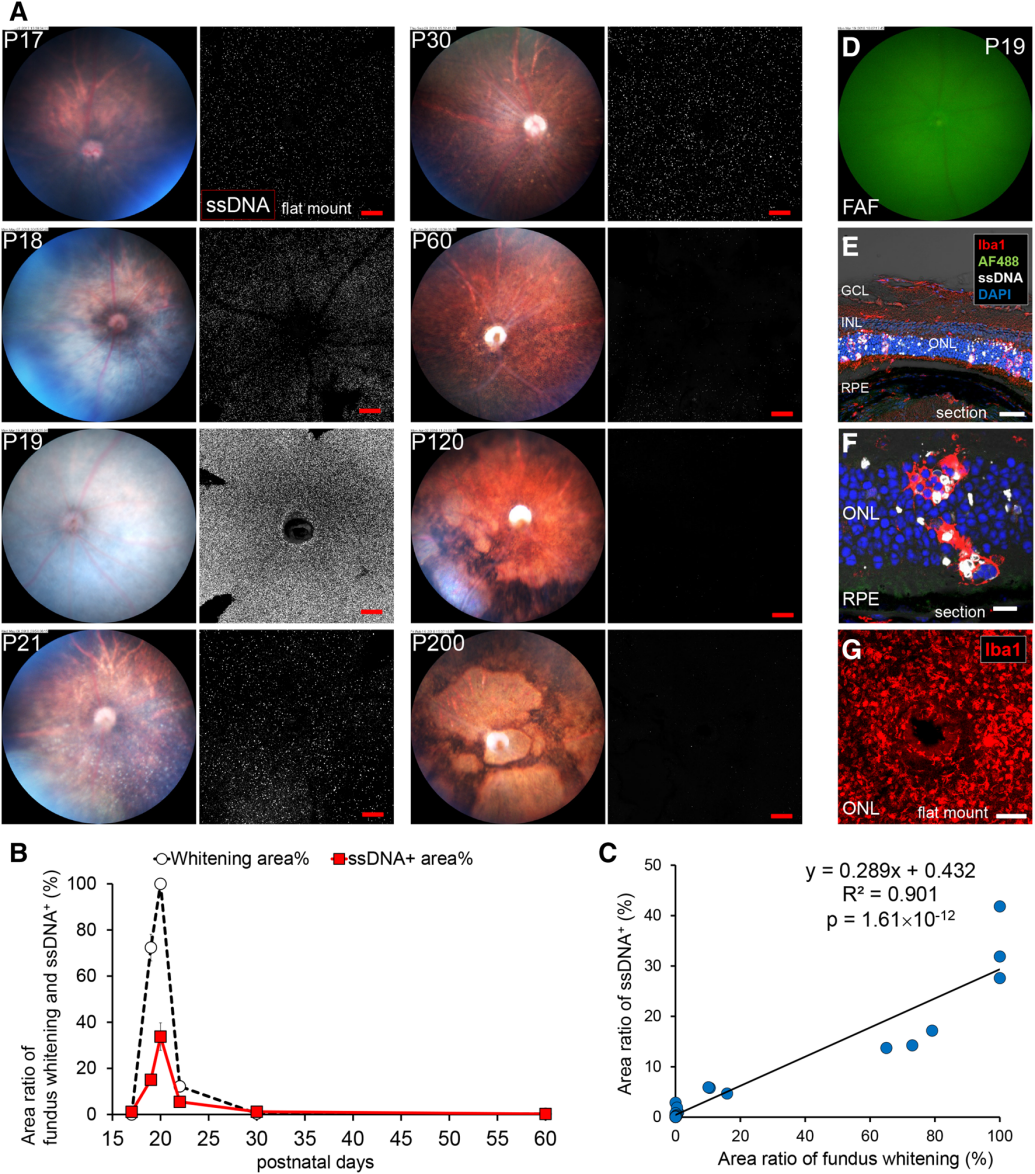
**Relationship between fundus whitening and apoptosis of photoreceptors in the retina of** ***rd10*** **mice.** (**A**) Pictures show the time course change of the fundus color and apoptosis in the retina of *rd10* from P17 to P200. Left pictures are in vivo fundus color photography. Right pictures show the confocal images of extracted flat-mounted retina at the ONL level, labeled with an apoptosis marker ssDNA (white). Scale bars, 100 μm. Fundus started to whiten on P19. Whitening spread throughout the whole surface on P20. On P22, white granular spots on the subretina were observed. Around P30–P60, the color tone of the retina became normal. Remarkable retinal atrophy was observed after P120. (**B**) Graphs show time course of fundus whitening area (%) (White marker) and ssDNA^+^ area (%) (Red marker). The markers of the graph show mean and standard deviation (SD) (n = 3). Peaks of both parameters coincide. (**C**) Scatter plot shows the relationship between the ssDNA^+^ area (%) and the whitening area (%). Measurement points were P17, 18, 19, 21, 30, 60, 120 and 200, n = 3 respectively. A linear relationship was found between both factors (R^2^ = 0.901). (**D**) There were no autofluorescent spots in FAF imaging of the central retina. (**E**, **F**, **G**) Pictures show the histopathological findings on fundus whitening at P19. (**E**) Flat-mounted retina labeled with Iba1, ssDNA and DAPI. SsDNA^+^ apoptotic photoreceptors in the ONL and infiltration of Iba1^+^ microglia from the ONL to the OS were observed. Scale bar, 40 μm. AF488, autofluorescence excited by 488 nm light; GCL, ganglion cell layer; INL, inner nuclear layer; RPE, retinal pigment epithelium. (**F**) Magnified image of the retinal section at the ONL. Microglia infiltrated from the ONL to the OS and phagocytized both apoptotic and non-apoptotic photoreceptors. Scale bar, 10 μm. (**G**) Retinal flat mount of *rd10* (P22) at the depth of the OS, labeled with Iba1. Iba1^+^ microglia accumulated in the OS at a high density. Scale bar, 100 μm.

In wild type (WT) C57BL/6 J ocular fundus, no change in retinal color tone or no hyperfluorescent spot by FAF was observed (Supplemental Fig. 1A). Ramified form of microglia resided in the inner and outer plexiform layers (IPL and OPL) in normal retina (Supplemental Fig. 1B). In the retinas of *rd10* mice, the whitish part was observed in color fundus photographs from postnatal 18 to 21 days (P18–P21) followed by multiple granular white dots at P21 (Fig. 1A). Increased FAF became evident from P19 and peaked at P22, which gradually decreased thereafter (Supplemental Fig. 2).

**Figure 2:**
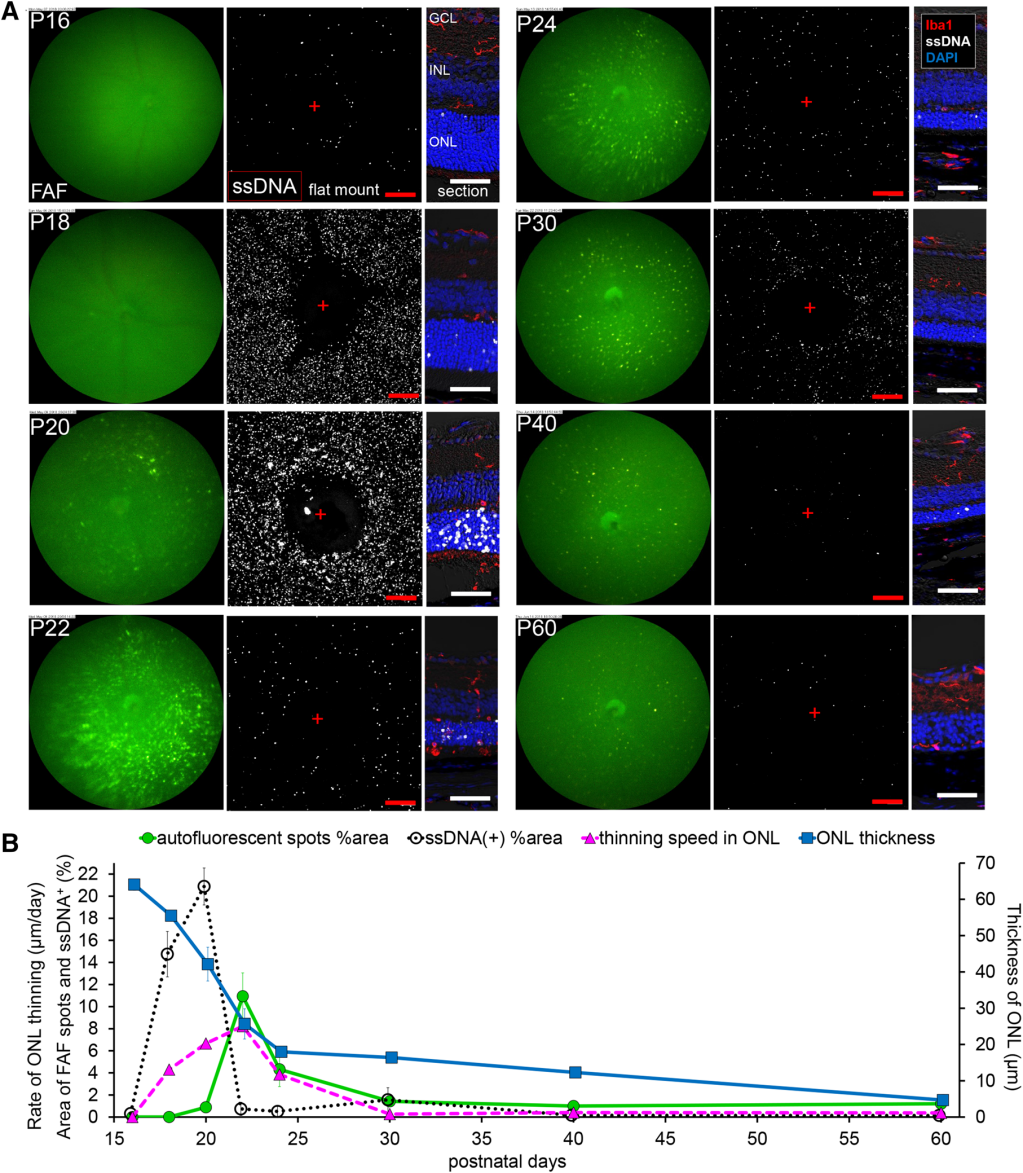
**Relationship in fundus autofluorescent spots, apoptosis of photoreceptors and retinal ONL thickness in the central retina of** ***rd10*** **raised under regular light cycle**. (**A**) Pictures show the time course change in the central retina of *rd10* from P16 to P60. Left pictures are in vivo FAF imaging with a retinal imaging microscope. Middle pictures show the confocal images of extracted flat-mounted retina at the ONL level, labeled with an apoptosis marker ssDNA (white). Scale bars, 100 μm, “ + ” show the center of the optic nerve head. Right pictures show the retinal section labeled with DAPI (blue), ssDNA (white) and Iba1 (red). Scale bars, 50 μm. (**B**) The graphs show the quantification of the FAF spot area % (green line), the ssDNA^+^ area % (black line), the thickness of the ONL (blue line) and thinning rate of the ONL (magenta line). The markers of the graph show mean and SD (n = 4). The peak of thinning rate of the ONL and the peak of FAF spot area were observed synchronously on P22 with a slight delay after the peak of apoptosis.

### Whitish retinas on color fundus was related to photoreceptor apoptosis

To investigate the relationship between the whitish fundus and the apoptosis of the photoreceptors in *rd10* mice retinas, fundus color photographs and distributions of apoptotic cells in isolated flat-mounted retina were compared during the degeneration process. In color fundus photographs, the entire fundus oculi became uniformly white on P19 (Fig. 1A). Similarly, the number of apoptotic cells that were labeled by an antibody against single strand deoxyribonucleic acid (ssDNA) peaked on P19. The occupancy of the whitish fundus and the apoptotic cells correlated well (Fig. 1B, C). The calculated regression line was as follows: (Area ratio of ssDNA^+^ %) = 0.289 (area ratio of whitish fundus %) + 0.432 (R^2^ = 0.901, *p* = 1.61 × 10^–12^).

To understand the pathological phase of the uniformly whitish fundus, histological examination was carried out on the retinal sections of *rd10* retinas at P19. At this time point, no autofluorescence appeared at the center of the fundus by FAF imaging (Fig. 1D). However, a considerable number of amoeboid-type microglia were infiltrating into the ONL and phagocytizing ssDNA labeled apoptotic photoreceptors. Some microglia were further migrating into the depth of the photoreceptor outer segments (OS) (Fig. 1E, F, G).

### Widespread diffuse distribution of apoptosis precedes increased FAF and substantial ONL loss in *rd10* mice

**Figure 3:**
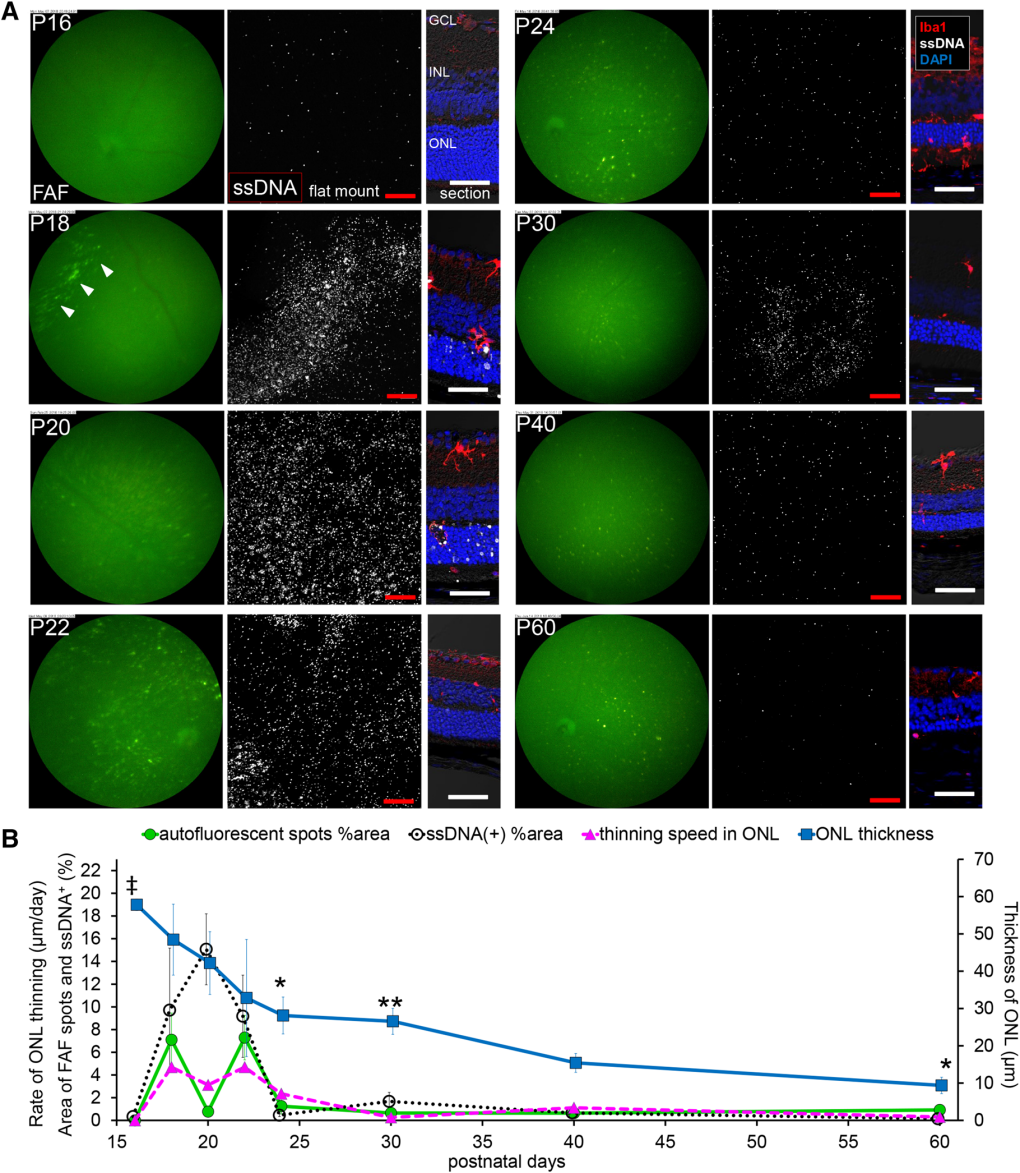
**Relationship in fundus autofluorescent spots, apoptosis of photoreceptors and retinal ONL thickness in the peripheral retina of** ***rd10*** **raised under regular light cycle.** (**A**) Pictures show the time course change in the peripheral retina of *rd10* from P16 to P60. Left pictures are in vivo FAF imaging with a retinal imaging microscope. Middle pictures show the confocal images of extracted flat-mounted retina at the ONL level, labeled with ssDNA (white). Scale bars, 100 μm. Right pictures show the retinal section labeled with DAPI (blue), ssDNA (white) and Iba1 (red). Scale bars, 50 μm. Arcuate FAF spots emerged on P18 (arrowhead). Arcuate aggregates of ssDNA^+^ cells also emerged on P18, and then subsequently appeared diffusely spread. (**B**) The graphs show the quantification of the FAF spot area % (green line), the ssDNA^+^ area % (black line), the thickness of the ONL (blue line) and thinning rate of the ONL (magenta line). The markers of graph show mean and SD (n = 4). (‡*p* < 0.01 (Center > Periphery), *p < 0.05, **p < 0.01 (Periphery > Center), *t* test of ONL thickness compared to the central retina). The area of autofluorescent spots showed a bimodal course. The first peak was from arcuate autofluorescent spots and the second peak was from diffuse autofluorescent spots. The bimodal peaks of the thinning rate of the ONL and the bimodal peaks of the FAF spot area were observed synchronously on P18 and P22.

To investigate the relations among FAF, apoptosis of the photoreceptors and ONL loss, the area with hyperfluorescence in FAF imaging, ssDNA positive cells in flat-mounted retina and ONL thickness in retinal sections were quantified and plotted over time in the central (Fig. 2) and the peripheral (Fig. 3) retinas (n = 4). In the central part of *rd10* retinas, widespread diffuse distribution of apoptotic photoreceptors indicated by ssDNA were observed on P18 and peaked on P20, while FAF spots with high intensity were detected from P20 and were most evident on P22 (Fig. 2A, B). The ONL thickness dramatically decreased by P24, and then the decrease slowed down. The FAF was maximum at the peak of ONL thinning. Altogether it was suggested that photoreceptor apoptosis precedes substantial ONL loss and the peak of FAF occupancy (Fig. 2B).

In the peripheral *rd10* retina, a characteristic arcuate apoptosis area indicated by ssDNA was observed on P18, followed by apoptosis in widespread diffuse area on P20 (Fig. 3A, B). By FAF imaging, an arcuate hyperfluorescent area was also observed on P18, followed by the appearance of widespread diffuse hyperfluorescent spots on P22, again suggesting that apoptosis of photoreceptors precede the emergence of hyperfluorescent FAF spots. The ONL thickness reduced more slowly in the peripheral part of the retina compared to the central part (Fig. 3B). The time course of FAF occupancy showed two-peaks that coincided with the peaks in the rate of ONL thinning (Fig. 3B).

### Diffuse cell apoptosis, diffuse FAF spots and ONL loss was significantly suppressed in *rd10* mice raised in dark

**Figure 4:**
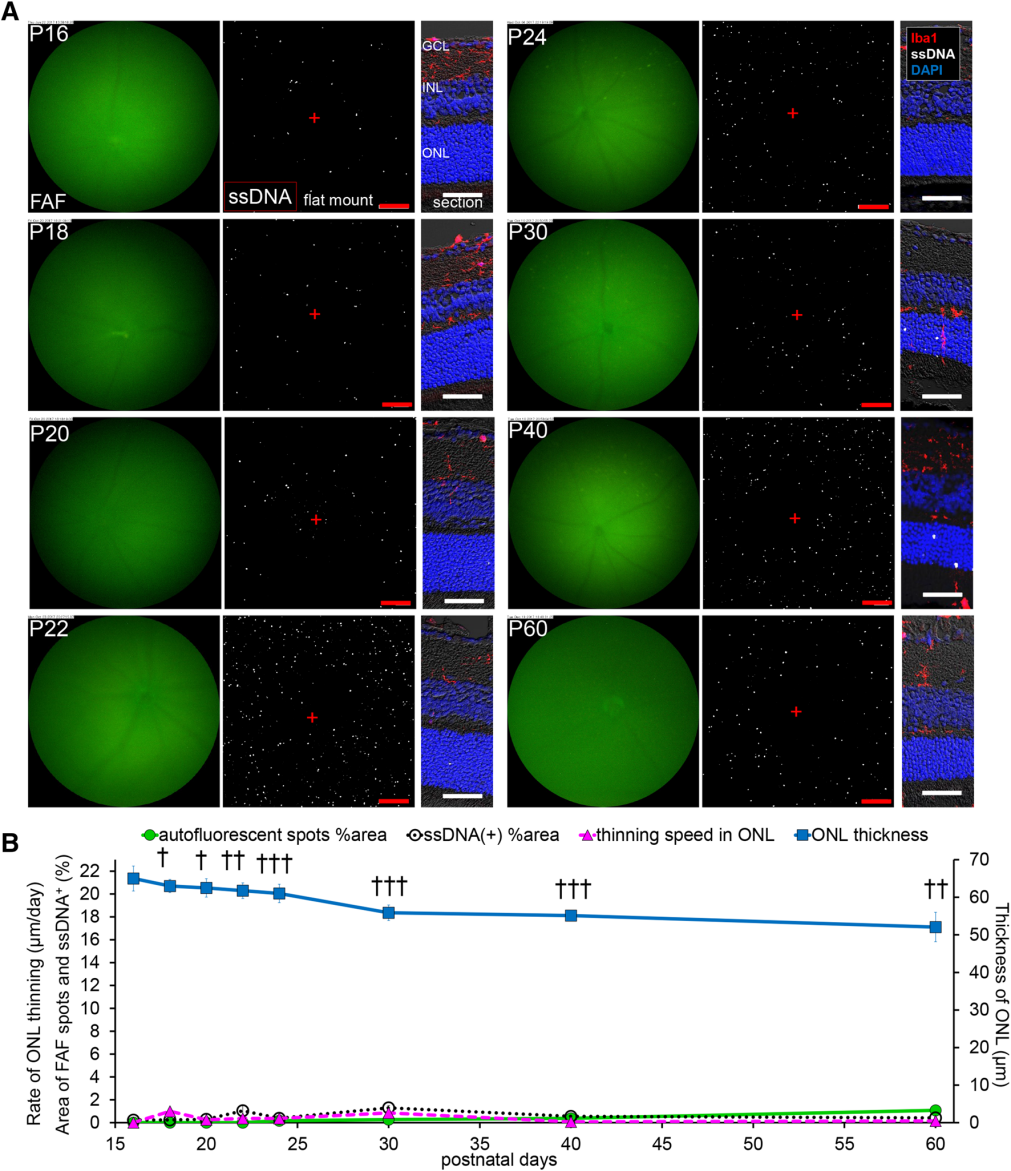
**Relationship in fundus autofluorescent spots, apoptosis of photoreceptors and retinal ONL thickness in the central retina of** ***rd10*** **raised in dark.** (**A**) Pictures show the time course change from P16 to P60, in the central retina of *rd10* mice raised in dark. Left pictures are in vivo FAF imaging with a retinal imaging microscope. Middle pictures show the confocal images of extracted flat-mounted retina at the ONL level, labeled with ssDNA (white). Scale bars, 100 μm, “ + ” show the center of the optic nerve head. Right pictures show the retinal section labeled with DAPI (blue), ssDNA (white) and Iba1 (red). Scale bars, 50 μm. (**B**) The graphs show the quantification of the FAF spot area % (green line), the ssDNA^+^ area % (black line), the thickness of the ONL (blue line) and the thinning rate of the ONL (magenta line). The markers of the graph show mean and SD (n = 4). (†*p* < 0.005, ††*p* < 0.00005, †††*p* < 0.00000005, *t* test in comparison to the central retina raised under regular light cycle). Thinning of ONL was significantly suppressed compared to the group raised under regular light cycle. The appearance of FAF spots and ssDNA^+^ apoptotic cells were also suppressed in dark-raised group.

**Figure 5:**
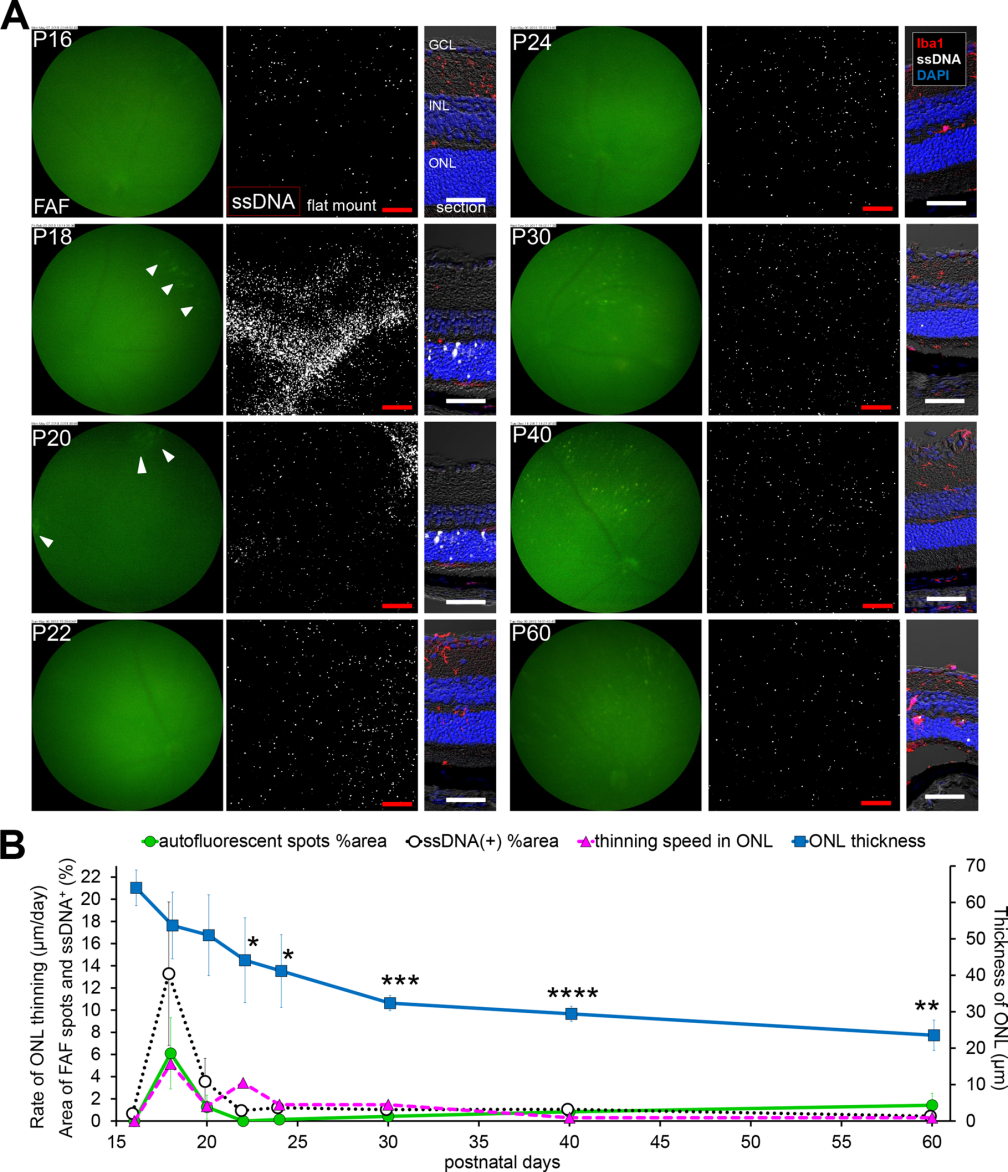
**Relationship in fundus autofluorescent spots, apoptosis of photoreceptors and retinal ONL thickness in the peripheral retina of** ***rd10* raised in dark.** (**A**) Pictures show the time course change from P16 to P60, in the peripheral retina of *rd10* raised in dark. Left pictures are in vivo FAF imaging with a retinal imaging microscope. Middle pictures show the confocal images of extracted flat-mounted retina at the ONL level, labeled with ssDNA (white). Scale bars, 100 μm. Right pictures show the retinal section labeled with DAPI (blue), ssDNA (white) and Iba1 (red). Scale bars, 50 μm. Arcuate FAF spots emerged from P18 to P20 (arrowhead). Arcuate aggregates of ssDNA^+^ cells also emerged from P18 to P20. (**B**) The graphs show the quantification of the FAF spot area % (green line), the ssDNA^+^ area % (black line), the thickness of the ONL (blue line) and thinning rate of the ONL (magenta line). The markers of the graph show mean and SD (n = 4). (*p < 0.05, **p < 0.0005, ***p < 0.00005, ****p < 0.000005, *t* test in comparison to the central retina raised in dark). The appearance of arcuate FAF spots peaked on P18. The peak of the thinning rate of the ONL, appearance of the FAF spots and apoptosis were observed synchronously on P18. Thinning of ONL in the peripheral retina was significantly more progressed compared to the central retina of the group raised in dark.

Since it has been known that retinal degeneration is delayed in *rd10* mice if raised in dark, we studied how FAF, cell apoptosis and ONL loss are affected by dark environment. When bred in dark, hyper-fluorescence by FAF imaging was not evident in the central retina up to P60. The apoptosis of photoreceptors indicated by ssDNA was also suppressed. The ONLs were significantly thick compared to those bred under regular light cycle (Fig. 4A, B). In the peripheral retina, interestingly, a characteristic arcuate localization of apoptotic cells was observed on P18, but the presence was transient (P18–P20) and no widespread diffuse apoptosis was observed. Likewise, an arcuate hyperfluorescent area was observed on P18 but the emergence of widespread diffuse FAF was mostly suppressed. The ONL thinning in the peripheral retina was less suppressed by dark environment compared to the central retina (Fig. 5A, B). However, unlike those bred under regular light cycle, peripheral FAF and cell apoptosis showed only one peak around P18, which was the time ONL thinning was most rapid (Fig. 5B).

We observed two types of FAF in the retina of *rd10* mice. Early arcuate hyperfluorescent area was observed on P18 in the peripheral *rd10* retina raised either under regular light cycle or in dark (Supplemental Fig. 3A), while widespread diffuse hyperfluorescent spots that peaked around P22 was observed in both central and peripheral *rd10* retina raised under regular light cycle but were markedly suppressed if raised in dark (Supplemental Fig. 3A). The total ONL loss during the time course (P16–P60) was in the order as: central retina raised under regular light cycle > peripheral retina raised under regular light cycle > peripheral retina raised in dark > central retina raised in dark, which seemed to correlate with the sum of the FAF area during the time course (Supplemental Fig. 3B). These results suggest that regarding the correlation between the FAF area and the type of photoreceptor apoptosis, the peripheral arcuate FAF may follow the initial event of early photoreceptor apoptosis which occurs regardless of the light condition, while widespread diffuse patterns of apoptosis and increased FAF are suppressed in dark environment, leading to ONL protection.

### Increased FAF was also observed in retinal degeneration with delayed onset

To investigate the effect of light on the progression of retinal degeneration in *rd10* mice, we examined the changes in FAF and cell apoptosis before and after changing the light environment. The *rd10* mice were kept in dark from birth to postnatal 10 months and then were moved to an environment with regular light cycle. Significant increase in ssDNA^+^ apoptotic cells was observed 7 days after moving to regular light cycle, which accompanied increase in FAF by in vivo imaging (Supplemental Fig. 4).

### A microglial wave spatiotemporally followed the initial apoptotic wave from center to periphery in early retinal degeneration

**Figure 6:**
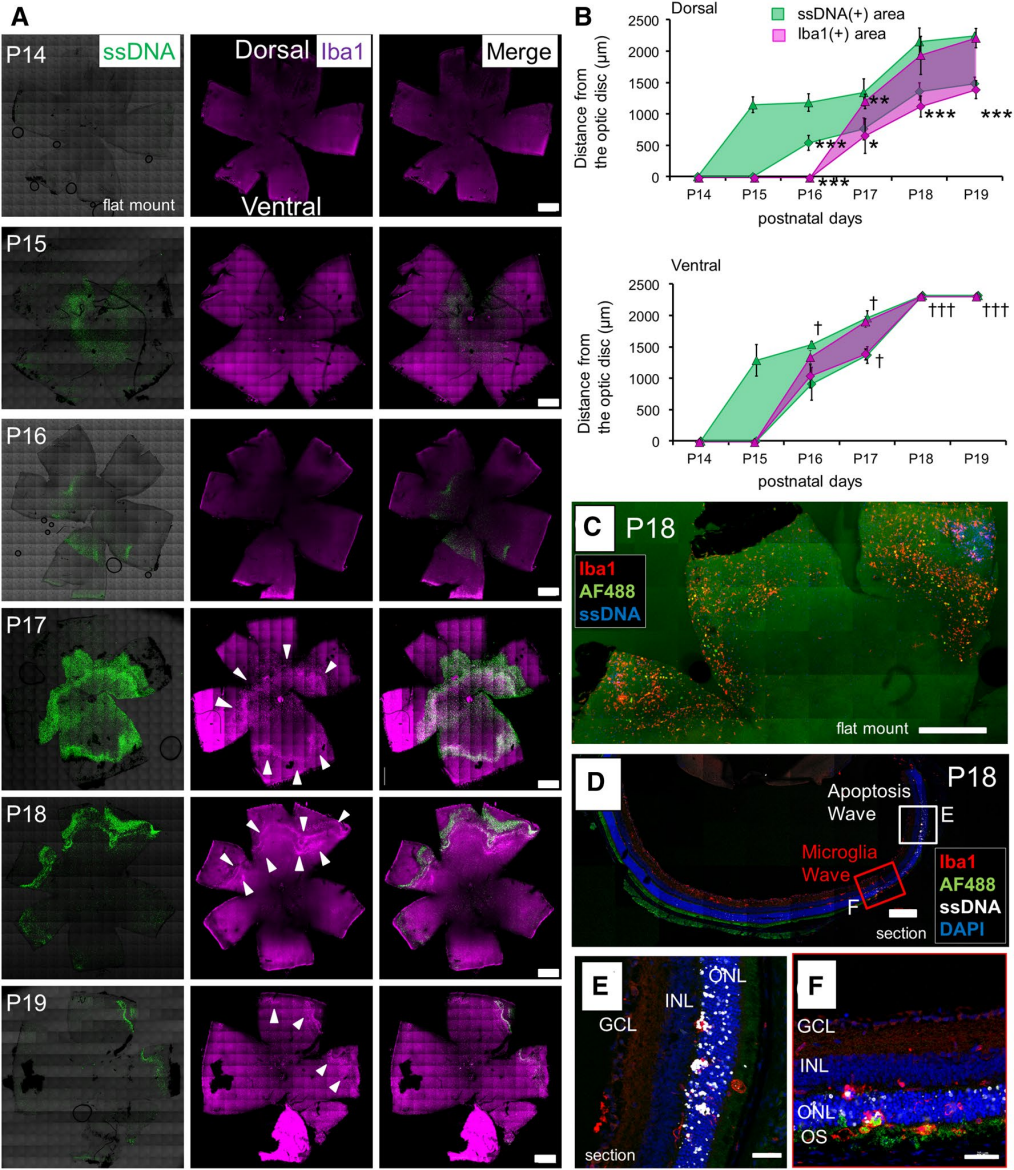
**Spreading wave of photoreceptor apoptosis, and autofluorescent arc formed by microglia following the wave.** (**A**) Retinal flat mounts in early phase of retinal degeneration in *rd10*, labeled with ssDNA and Iba1. The wave of photoreceptor apoptosis spread over days, and the wave of Iba1^+^ microglia followed the apoptotic wave. Scale bars, 500 μm. (**B**) Graphs show the inner and outer diameter of ssDNA^+^ apoptotic wave and Iba1^+^ microglia wave from the papillary center. The markers of the graph show mean and SD (n = 3, *p < 0.05, **p < 0.005, ***p < 0.0005, *t* test of the Iba1^+^ wave in comparison to the ventral site. †*p* < 0.05, †††*p* < 0.0005, *t* test of the ssDNA^+^ wave in comparison to the dorsal site). (**C**) Immunohistochemistry of flat-mounted retina of *rd10* (P18) showing positional relationship of apoptosis wave, microglia wave and autofluorescent spots. Some of Iba1^+^ microglia emitted autofluorescence. Scale bar, 500 μm. (**D**–**F**) Retinal section of *rd10* (P18) labeled with Iba1 and ssDNA. (**D**) The retinal section revealed positional relationship of apoptotic wave, microglia wave and autofluorescent spots. Scale bar, 200 μm. (**E**) Magnified image of the retinal section with apoptotic wave. Apoptosis of the photoreceptors are profoundly accumulated in the ONL. Scale bar, 40 μm. (**F**) Magnified image of the retinal section with microglia wave. Infiltration of many microglia into the ONL was observed. Microglia that reached the OS emit autofluorescence. Scale bar, 40 μm.

Arcuate autofluorescent spots and arcuate aggregation of apoptotic cells were observed in the peripheral retina of *rd10* mice at P18 under either regular light cycle or continuous dark (Figs. 3A, 5A). Earlier events preceding this phenomenon were sought by examining the spatiotemporal distributions of apoptotic cells and microglia in flat-mounted *rd10* retinas from P14 to P19. An initial wave of ssDNA^+^ apoptotic cells appeared on P15 and spread from the central towards the periphery. The wave of Iba1^+^ microglia appeared on P16–17 in the mid-peripheral retina and then moved towards the periphery following the wave of apoptosis (Fig. 6A). The spreading of apoptotic and microglial waves was significantly faster on the ventral side than the dorsal side (Fig. 6B). In P18 flat-mounted retina, some of the Iba1^+^ microglia were phagocytizing cells with autofluorescence at 488 nm excitation wavelength, whose localization corresponded to the peripheral arcuate FAF observed in Figs. 3 and 5 (Fig. 6C). In P18 retinal section, the peripheral apoptotic area showed many ssDNA^+^ apoptotic cells exclusively in the ONL (Fig. 6D, E). The area where microglia presented showed a lot of Iba1^+^ microglia infiltrating into the ONL and reaching to the OS where they showed autofluorescence (Fig. 6D, F). These results suggested that the peripheral arcuate FAF may indicate the event of initial apoptotic wave in the early phase of retinal degeneration in *rd10* mice, which occurred regardless of the light condition.

### Infiltration of phagocytic microglia into the layers of photoreceptors (ONL and OS)

In the retina of *rd10*, microglia exhibited two forms: ramified type and amoeboid type. Ramified type microglia were negative for a phagocytic marker CD68. Amoeboid type microglia were positive for CD68 (Supplemental Fig. 5A). The ramified form of microglia resided in the inner and outer plexiform layers (IPL and OPL) in normal retina. In the *rd10* retina, microglia did not show any change at P16. From P18 to P22, some microglia became CD68^+^ amoeboid type, infiltrated toward the ONL, and passed through the ONL to reach the OS. From P30 to P60, the expression of CD68 in microglia subsided (Supplemental Fig. 5B).

### Accumulated microglia in the subretinal space are the candidate source of diffuse FAF spots

**Figure 7:**
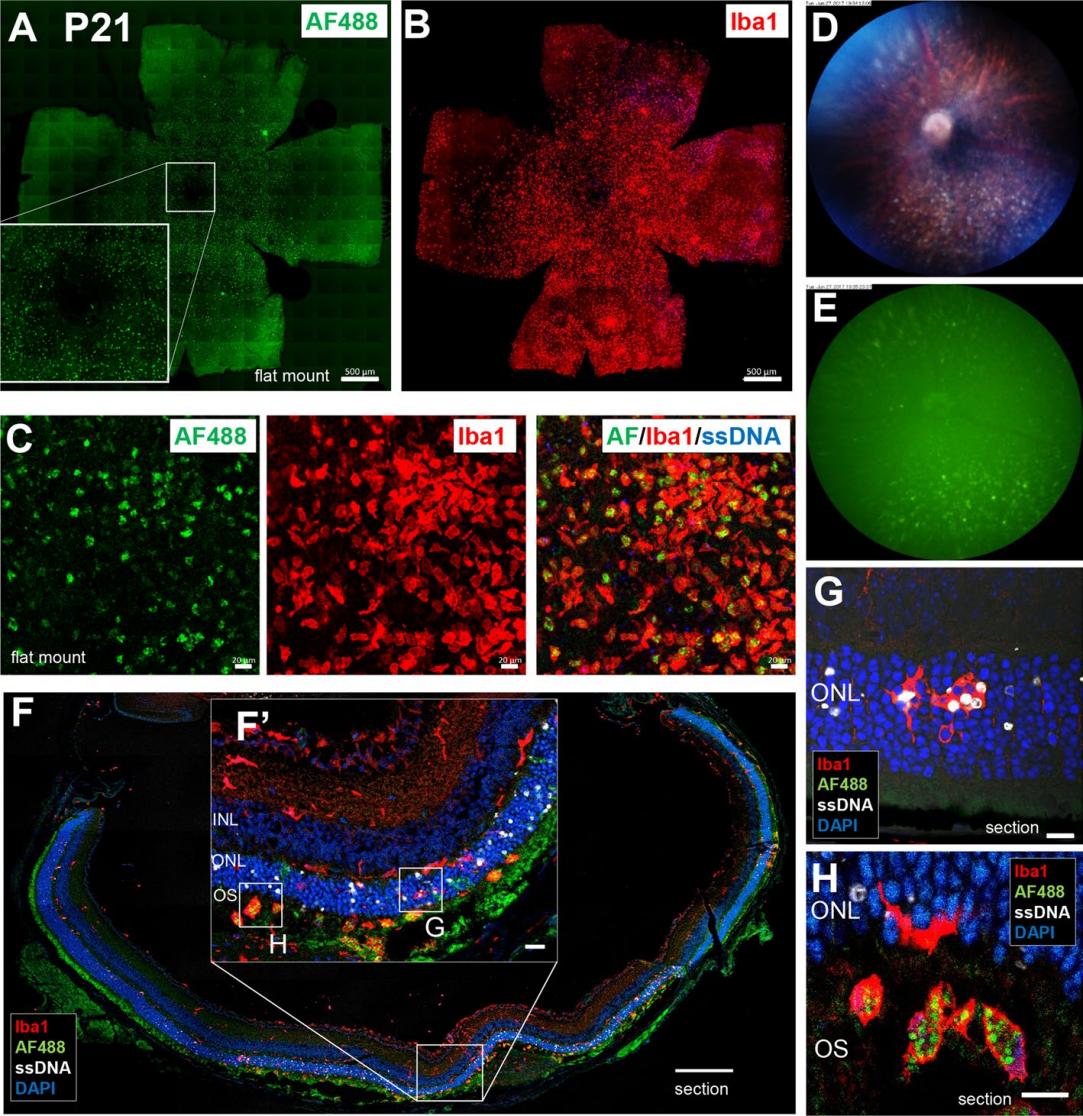
**Histopathological findings on diffuse fundus autofluorescent spots in the retina of** ***rd10*** **at P21.** (**A**) Accumulation of FAF spots was observed in the ONL around the central retina of *rd10* at P21. Scale bar, 500 μm. (**B**) Accumulation of Iba1^+^ microglia was observed in the ONL layer around the centeral retina of the same mouse. Scale bar, 500 μm. (**C**) Flat-mounted retina of *rd10* at P21 showing autofluorescent spots were labeled with Iba1 and ssDNA. The autofluorescent spots coincided with Iba1^+^ microglia but not with ssDNA^+^ apoptotic cells. Scale bars, 20 μm. (**D**) In the color fundus photograph, white granules accumulated under the retina were seen on P21. (**E**) In vivo FAF imaging of the same *rd10* mouse. The localization of the fluorescent spots coincided with white granules in the subretina. (**F**) Retinal section of *rd10* at P21 labeled with Iba1, ssDNA and DAPI. More apoptotic photoreceptors were observed in the ONL around the central retina. Scale bar, 200 μm. (**F′**) In retinal section near the center, Iba1^+^ microglia accumulated in the OS. Scale bar, 20 μm. (**G**) In the magnified image of the ONL, microglia that infiltrated into the ONL phagocytosed ssDNA positive and negative photoreceptors. Scale bar, 10 μm. (**H**) In the magnified image of the subretina, microglia in the OS stored fluorescent granules in their body. Scale bar, 10 μm.

Since widespread diffuse hyperfluorescent spots by FAF were most evident around P22 under normal light condition, histological examination was performed on *rd10* retina isolated at P21. In the flat-mounted central retina, the overall autofluorescence pattern was similar to the accumulation pattern of the Iba1^+^ cells (Fig. 7A, B). The immunohistochemistry (IHC) data showed that 488 nm autofluorescence colocalized with Iba1^+^ microglia but not with ssDNA^+^ apoptotic cells (Fig. 7C). In the fundus of *rd10* mice on P21, diffuse white spots on the retina were seen by color fundus photograph (Fig. 7D), and these spots emitted autofluorescence at 488 nm excitation wavelength (Fig. 7E). In the retinal section, a number of Iba1^+^ microglia with autofluorescence were observed in the OS layer (Fig. 7F, F'). In the ONL, amoeboid microglia with or without phagocytosed ssDNA^+^ and ssDNA^−^ photoreceptors were observed, but these microglia in the ONL did not present autofluorescence (Fig. 7G). On the other hand, microglia that reached the OS contained granules with 488 nm autofluorescence in their cell body (Fig. 7H, the substance of the 488 nm autofluorescence will be discussed later). Our results suggest that these microglia accumulated in the OS layer may contribute to the diffuse FAF spots.

### Apoptotic photoreceptors express the chemokine, fractalkine

**Figure 8:**
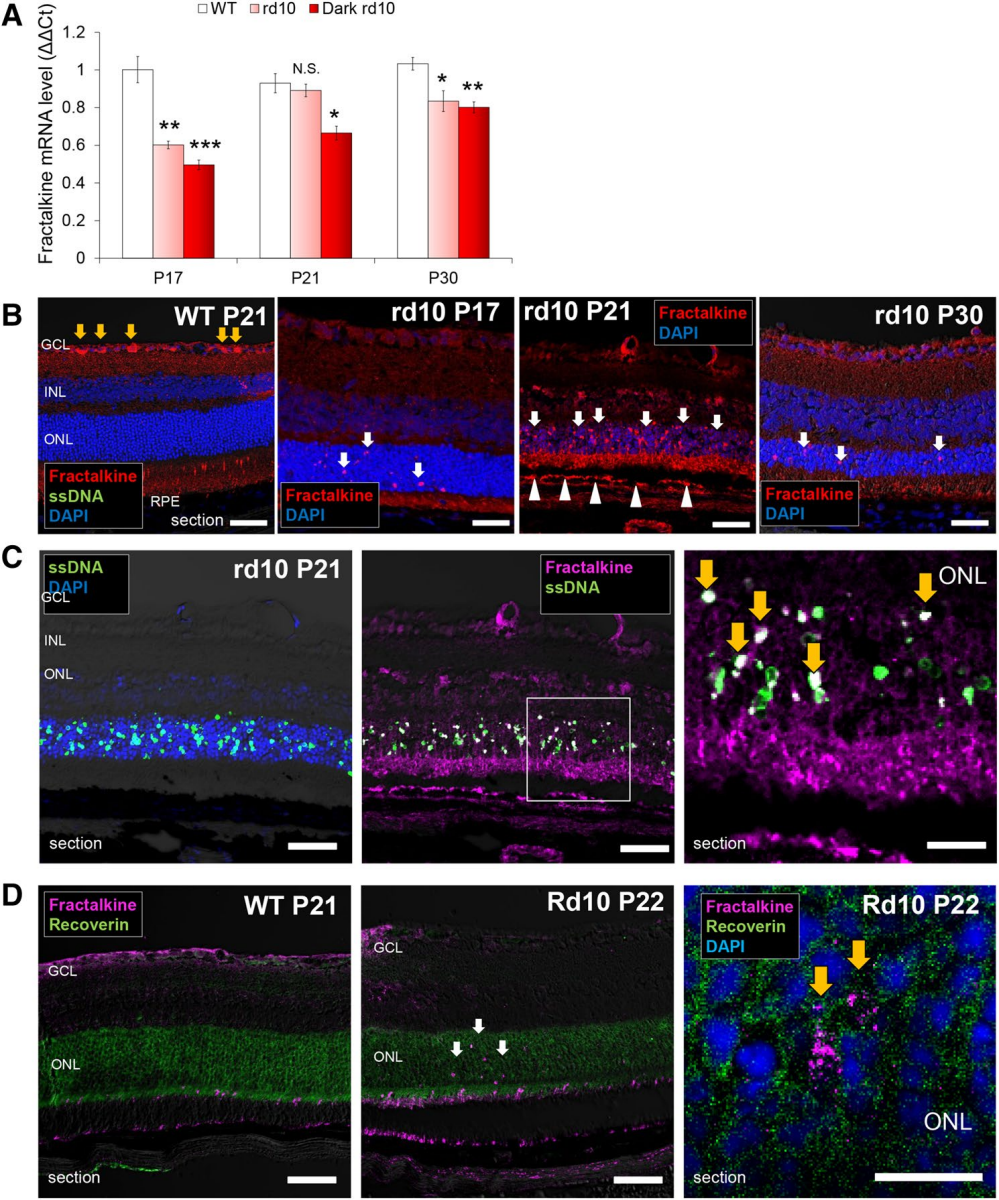
**Expressions and localizations of fractalkine in the retina of** ***rd10*** **mice.** (**A**) Comparison of the mRNA expressions of fractalkine in the retinas of WT C57BL/6 J, *rd10* raised under regular light cycle and *rd10* raised in dark, by qRT-PCR. Results indicate the relative expression (ΔΔCR: WT P17 = 1). The graphs show mean and SD of three experiments (*p < 0.05, **p < 0.005, ***p < 0.0005, ****p < 0.00005 compared to WT). N.S., not significant. (**B**) Retinas of WT (P21) and *rd10* (P17 to P30) labeled with fractalkine. In WT retina, fractalkine^+^ cells were found only in the GCL (yellow arrows). In the retina of *rd10*, obvious fractalkine^+^ cells were not found in the GCL but in the ONL (white arrows). On P21, fractalkine was also positive in the RPE (white arrowheads). Scale bars, 50 μm. (**C**) Retina of *rd10* at P21 labeled with ssDNA and DAPI (left), and with fractalkine and ssDNA (middle). Magnified image of the ONL in the middle picture (right). SsDNA^+^ cells scattered within the ONL and the localization of some ssDNA^+^ cells coincided with the staining of fractalkine (yellow arrows). Scale bars, (left and middle) 50 μm, (right) 20 μm. (**D**) Retinas of WT (P21) and *rd10* (P22) labeled with fractalkine and a photoreceptor marker recoverin. In WT retina (left) fractalkine^+^ cells did not coincide with recoverin-positive photoreceptors. In the retina of *rd10* (middle), fractalkine^+^ cells coincided with recoverin-positive photoreceptors (white arrows). In the magnified image of the ONL in *rd10* (right), granular staining of fractalkine was observed (yellow arrows). Scale bars, (left and middle) 50 μm, (right) 10 μm.

To investigate whether major chemokines are involved in the chemotaxis of microglia in this RP model, qRT-PCR for fractalkine/CX3CL1 and MCP-1/CCL2 was performed on the extracted retina of *rd10* mice at 3 different time points during retinal degeneration (P17, 21, and 30). The expression of fractalkine mRNA was lower in *rd10* retinas compared to WT except for P21 that showed the expression comparable to WT (Fig. 8A). MCP-1 mRNA was significantly higher in *rd10* retina than in WT at P21 and P30 (Supplemental Fig. 6A).

By IHC, fractalkine^+^ cells were found in the ONL as well as in the RPE layer of *rd10* retina at P21, while it was not detected either in the ONL or the RPE layer in WT (Fig. 8B). The staining of fractalkine colocalized with some of the ssDNA^+^ apoptotic photoreceptors in the ONL of *rd10* retinas (Fig. 8C). The staining of fractalkine colocalized with the area positive for a photoreceptor marker recoverin in the ONL of *rd10* retinas (Fig. 8D). These may suggest localized expression of fractalkine in some apoptotic photoreceptors, although the total gene expression level showed no difference compared to WT.

By IHC, MCP-1 expression was not detected in WT or *rd10* retina at P17 and P21. However, on P30, MCP-1 positive cells were observed in the outer retinal layer (Supplemental Fig. 6B), and some of the Iba1^+^ microglia were labeled with MCP-1 at the outer retina (Supplemental Fig. 6C), indicating some of the active microglia that migrated to the outer retina was MCP-1 positive.

### Contribution of RPE cells to fundus autofluorescence

It has been assumed that RPE cells, another type of retinal phagocyte, are involved in the FAF in RP patients^3^. Therefore, we examined the autofluorescence of RPE cells histologically. RPE is a monolayer of hexagonal black pigment cells. RPE cells in *rd10* mice were apparently similar to those in WT at P18, but on P22 the central RPE cells of *rd10* presented dim autofluorescence behind the brighter microglial autofluorescent spots, and showed abnormal morphologies including large size and irregular shapes (Supplemental Fig. 7A, B middle). On P30 they returned to a hexagonal shape, but some RPE cells contained autofluorescent granules in their cell bodies (Supplemental Fig. 7A, B right), which looked similar to those in microglia that contained the autofluorescent granules in CD68^+^ phagosomes (Supplemental Fig. 7C). In the peripheral, although microglia showed autofluorescence as in the central, RPE cells kept the hexagonal shape and did not show autofluorescence (Supplemental Fig. 7A). Altogether, the RPE cells in the central but not the peripheral retina of *rd10* were active like microglia, changing their morphologies and accumulating autofluorescent granules in their cell bodies. These results suggest that RPE cells together with microglia contribute to the formation of FAF in RP patients.

## Discussion

Whitening and autofluorescent spots were observed on the fundus of *rd10* mice. The extent of retinal whitening linearly correlated with the number of apoptotic photoreceptors. Although the time points of the appearance of the autofluorescent spots were different between the central and the peripheral retinas, or whether raised under regular light cycle or in dark, the peak period of FAF appearance overlapped with the peak period of retinal ONL thinning. We therefore hypothesized that microglia that received signals from apoptotic photoreceptors in degenerative retina of RP could migrate into the ONL and contribute to the development of FAF, thus be a biomarker to indicate the progression of RP. In the present study, we obtained the findings that support the hypothesis as follows: (1) Histopathological examination revealed that microglia that phagocytosed apoptotic cells and migrated into the OS layer were the source of autofluorescent spots, (2) From the fundus whitening and autofluorescence intensity, we were able to know the severity of photoreceptor apoptosis and the thinning rate of the ONL, in other words the severity of the ongoing retinal degeneration, and (3) In the retinas of *rd10* mice, fractalkine was expressed in apoptotic photoreceptors unlike those of WT. We speculate that fractalkine from apoptotic photoreceptors may induce microglial infiltration into the ONL in *rd10* mice.

The retina of *rd10* on P20 became turbid white. On the next day it turned into white spots with autofluorescence, and those spots were microglia. This suggests microglia are also involved in fundus whitening. The retina is whitened by commotio retinae due to ocular contusion. According to the report of histopathological examination on commotio retinae, a rupture in the OS has been observed^14^. In the retina of *rd10* on P20, a large number of microglia infiltrated and reached the OS. Considering this observation together with the report on commotio retinae, we speculate that scattering of reflected light by microglia arriving at the OS, or phagocytosis of the OS by microglia, are related to retinal whitening. Apoptosis of photoreceptors and infiltration of microglia into the ONL has been known to correlate^10^, which is consistent with the results of this study showing that microglia may be involved in fundus whitening and that fundus whitening is correlated with the amount of photoreceptor apoptosis.

In the present study, we observed two patterns of cell apoptosis associated with respective patterns of FAF. Arcuate FAF was formed by early microglial wave following the initial wave of photoreceptor apoptosis spreading from the center to the periphery at the onset of retinal degeneration in *rd10* mice (P15–P18). Some microglia that reached the OS emitted autofluorescence in the peripheral retina on P18, which was observed in *rd10* mice raised either under regular light cycle or in dark. On the other hand, widespread diffuse FAF peaked around P22 following widespread diffuse apoptosis that peaked around P19–P20. This diffuse pattern was markedly suppressed in *rd10* mice raised in dark. Microglia that phagocytized diffusely distributed dying photoreceptors and then reached the OS contributed to the diffuse hyperfluorescent spots. In the retina of RP model mice, the number of microglia infiltrating into the ONL correlates with the number of apoptotic photoreceptors^10^. Together with our results, this suggests that the appearance of FAF spots reflects how much apoptosis has occurred. The short wave FAF signal is known to originate from bisretinoid lipofuscin that is formed in photoreceptor cells as a product of visual cycle reactivity^15,16^. We found that microglia reaching the OS layer became autofluorescent. The emergence of phagocytic microglia/macrophage producing autofluorescence in diseased brain^17^, spinal cord^18^, and retina^6^ have been reported. The origin of the autofluorescence of microglia/macrophage has been reported to be redox cofactors such as flavin adenine dinucleotide^19^ or lipofuscin granules^20^. Fluorescent materials in the photoreceptors of the OS layer, which were phagocytosed by microglia, were considered to be the origin of the FAF spots. Taken together, we newly showed that microglia along with RPE cells contributed to the formation of FAF that reflect the type and the extent of photoreceptor apoptosis in RP.

In *rd10* mice, retinal degeneration seemed to occur with initial wave of apoptosis followed by widespread diffuse apoptosis. In the early stage of retinal degeneration (P15–P19), the wave of photoreceptor apoptosis spread from the center to the periphery. Since this initial apoptotic wave appeared also in the group raised in dark, the light environment might not affect the initial apoptotic wave. On the other hand, widespread diffuse apoptosis was suppressed if raised in dark, and under this condition thinning of the peripheral ONL was severe but the central part was mostly preserved, while under regular light cycle, widespread diffuse apoptosis occurred and degeneration of the central ONL was severe. This indicates the difference of characteristics between the two types of apoptosis: the initial apoptotic wave is independent of the light condition and is involved exclusively in the degeneration of the peripheral ONL, while the later diffuse apoptosis depends on the light condition and is involved in the degeneration of the central ONL. Visual signal triggered by rhodopsin activation is transduced via *PDE6* that hydrolyzes cGMP in the rod photoreceptors. Mutation of *PDE6β*, one of the catalytic units in *PDE6*, in *rd10* mice results in elevated cGMP in the rod photoreceptors^21^, opened cGMP-gated channels and increased intracellular calcium, which leads to apoptosis^22^. It could be possible that photo damage to fragile *PDE6β-*mutated photoreceptors accelerated the widespread diffuse apoptosis as shown in this study. Similar to the *rd10* mice, shading may be effective for retinal protection in some types of RP patients who have mutation in *PDE6β*.

During the course of degeneration in the retina of *rd10*, the peak of ONL thinning overlapped with the peak of FAF appearance. Total FAF area over time correlated with the total thinning of the ONL in each group. Genetic ablation of microglia and inhibition of microglial phagocytosis ameliorate photoreceptor degeneration in *rd10* mice^10^. This together with the results of our study suggest that recruitment of microglia triggered by apoptosis may further accelerate the ONL loss. Since microglia that infiltrated through the ONL became fluorescent when they reached the OS, the progress of photoreceptor phagocytosis can be estimated by FAF intensity. It is known that hyperautofluorescent ring appears in the fundus of human RP patients. As the retinal degeneration progresses, the hyperautofluorescent ring narrows^3,23^. The hyperautofluorescence corresponds to the boundary of degeneration as revealed by OCT imaging^2^. These together with the results of our study suggest FAF should represent the ongoing ONL loss.

Although the mRNA expression of fractalkine in the retina of *rd10* was not increased compared to WT, immunohistological examination suggested the expression of fractalkine by some apoptotic photoreceptors. In the retina of *rd10*, microglia expressed CD68, a lysosomal marker indicative of phagocytic activity of microglia^24^, and infiltrated into the layers of photoreceptors (ONL and OS). Microglia expresses the receptor of fractalkine, CX3CR1^25^. In normal retina, fractalkine is expressed in the ganglion cell layer (GCL) and the INL, which coincide with the normal existence site of microglia^26^. Fractalkine is suggested to acts as a “find-me signal”, recruiting microglia toward apoptotic cells to promote clearance of the apoptotic cells by phagocytic microglia^27^. In a retinal light-damage model, fractalkine is expressed in apoptotic photoreceptors and attract microglia to the injured ONL^11^. In *rd10* mice, fractalkine signal negatively regulates phagocytosis of microglia^28^. These reports together with the results of our study suggest that overall decrease of fractalkine expression in the retina of *rd10* activates microglia and altered fractalkine distribution directs the microglia to apoptotic photoreceptors to clear them. On the other hand, as shown in this study, upregulation of MCP-1 had a time lag from the peak of apoptosis or the appearance of FAF spots. Histologically, MCP-1 was expressed in some microglia that migrated into the outer retina. Activated microglia are able to produce MCP-1^5,12^. The increased expression of MCP-1 in the retina of *rd10* may be the consequence of microglia activation after retinal degeneration, leading to neuroinflammation.

We admit there are several limitations in this study. In *rd10* mice, the progression speed of retinal degeneration is faster than human RP and the appearance pattern of FAF is different from human RP patients. In this study, we showed the arcuate FAF that appears during the early stage of the degeneration in *rd10* mice resembles the hyperfluorescent ring in the fundus of human RP patients in terms of the fact that both demarcates the degeneration area of the photoreceptors. We also showed the involvement of microglia in the FAF formation of *rd10* mice. However, it is unclear from the results of this study whether microglia contribute to the hyperfluorescent ring in human RP patients. Successful imaging of microglia in the human retina by adaptive optics imaging has been reported^29^. In the future, it may be possible to get direct evidence with adaptive optics whether microglia contribute to the FAF of human RP patients.

The reason of the difference in the phenotype of FAF between *rd10* mice and human RP patients may be the differences in spacial distribution of the rod/cone^30^, the presence or absence of macular structure, the speed of degeneration, and the size of the eye. However, an observation in *rd10* mice is significant to interpret human fundus imaging of RP patients because the functional interactions between the retinal cells such as the photoreceptor cells, retinal microglia, and RPE cells should share much in common with those of human.

In conclusion, the fundus whitening and FAF spots were observed in *rd10* mice during the course of degeneration. The fundus whitening correlated with apoptosis of the photoreceptors, and the area of FAF spots indicated the thinning rate of the ONL. Thus, fundus whitening and FAF spots can be an indicator for the ongoing severity of degeneration in RP model mice. If the involvement of microglia in the fundus color and autofluorescence is also applicable to human RP, analyzing the fundus color and FAF in detail may help to read the ongoing severity of degeneration in RP patients as well. Histologically, microglia that became phagocytes contributed to FAF formation. The localization of fractalkine to apoptotic cells was suggested to contribute to microglia migration into the ONL. The present results highlight the mechanism and the potential of fundus imaging that might be an indicator of the ongoing disease severity in RP patients.

## Methods

### Experimental animals

All animal experiments were performed according to the guidelines for animal experiments of RIKEN Center for Biosystems Dynamics Research and approved by the Animal Experiment Committee of the RIKEN Kobe Institute (Approval ID: A2006-05-40). All procedures complied with the ARVO Statement for the Use of Animals in Ophthalmic and Vision Research. WT C57BL/6 J mice were obtained from CLEA Japan, Inc. PDE6β^rd10^/J (*rd10*) mice were obtained from Jackson laboratory, ME, USA and they were housed at a local animal facility under standard laboratory conditions (18–23 °C, 40–65% humidity, and a 12 h light–dark cycle) with free access to food and water throughout the experimental period. A dark-raised group was raised in a dark place for 24 h as a treatment intervention for *rd10*.

### Immunohistochemistry

The extracted eyeballs were fixed with 4% paraformaldehyde. Retinas were peeled from the eyeball to prepare flat-mounted specimens. Eyeballs for sectioning were embedded in OCT compound (Sakura Finetek Japan, Tokyo, Japan). Frozen blocks were made at −  80 °C and sections of 10 μm thickness were made using HM 560 CryoStar cryostat (Thermo Fisher Scientific, Waltham, MA). Specimens were blocked in 10% donkey serum in 1 × PBS with 0.3% Triton X-100 for 1hour at room temperature and then incubated with primary antibodies in 1 × PBS with 0.3% Triron X-100 for 24 h at 4 °C (retinal section) or for 5 days at 4 °C (retinal flat mount). Primary antibodies included goat anti-Iba1 (× 150, Novus, Centennial, CO, USA), rabbit anti-Iba1 (× 1,000, Wako, Osaka, Japan), rat anti-CD68 (× 500, BioLegend, San Diego, CA, USA), goat anti-fractalkine (× 100, SantaCruz, Dallas, TX, USA), rat anti-MCP-1 (× 100, Novus), rabbit anti-recovrerin (× 1,000, Proteintech, Rosemont, IL, USA) and rabbit anti-ssDNA (× 50, IBL, Fujioka, Japan). The ssDNA is a specific marker for apoptosis^31^. After washing in 1 × PBS with 0.3% Triton X-100, retinas were incubated for 1 h (section) or overnight (flat mount) with the secondary antibody (Alexa Fluor-488 conjugated donkey anti rabbit IgG; Alexa Fluor-546 conjugated donkey anti rabbit, goat IgG; Alexa Fluor-647 conjugated donkey anti rabbit or rat IgG) and DAPI (× 1,500 Sigma-Aldrich, Dt. Louis, MO, USA). Images were acquired with a confocal microscope LSM700 (Zeiss, Oberkochen, Germany).

### In vivo* fundus imaging*

After the pupils of mice were dilated with 0.1% Phenylephrin and 0.1% Tropicamid the mice were sedated by inhalation of 1% isoflurane delivered by a nose cone. In vivo shortwave fundus autofluorescent and color images were obtained using the scanning laser ophthalmoscopy; The Micron IV Retinal Imaging Microscope (Phoenix Research Labs, CA). The field of view as measured from the entrance pupil will be above 50°. The short wave FAF image was obtained by irradiating blue light as excitation light to the retina and separating fluorescence with a 500 nm barrier filter.

### Imaging analysis

Images were analyzed with imaging processing software ImageJ^32^. (1) Color fundus photography: The area of fundus whitening at each day of age were measured. The ‘color threshold’ was adjusted to a whitish part, and the %area was calculated using the ‘analyze particle’ function of ImageJ. (2) FAF imaging: Images were converted into 8-bit images. In raw FAF image, the background is brighter at the center than the periphery and hinders the analysis of the fluorescent spot. For this analysis, the background was excluded with rolling ball function (radius 50 pixels) to leave the fluorescent spot. ‘Threshold’ was adjusted to the FAF spots and the %area was calculated using the ‘analyze particle’ function of ImageJ. The area of FAF spots in the central retina at each day of age were measured in the region of 100 × 100 pixels on the dorsal area within the distance of 2 times of papillary diameters from the optic disc. The area of FAF spots in the peripheral retina were measured in the region of 100 × 100 pixels on the dorsal area at the distance of more than 6 times of papillary diameters apart from the optic disc. If arcuate FAF spots appeared in the peripheral retina, the area was measured in the arcuate FAF spots region. (3) Flat-mounted retina: In the analysis of apoptosis, the area of ssDNA positive part of immunostaining in retinal flat mount at each day of age were measured. Images were converted into 8-bit images. ‘Threshold’ was adjusted to ssDNA^+^ parts and the %area was calculated using the ‘analyze particle’ function of ImageJ.

### Quantitative RT-PCR

Total RNA from retinas was extracted using an RNA isolation kit (Roche, Basel, Switzerland) and reverse transcribed to generate cDNA with Transcriptor First Strand cDNA Synthesis kit (Roche). For qPCR, cDNA was amplified with a LightCycler 480 system (Roche) using a qRT-PCR master mix (Roche), Universal Probe Library primers, and probes (Roche)^33^. The qRT-PCR was performed as per a previous report^33^. The primers and probes were as follows: *Fractalkine/Cx3Cr1*, forward primer, 5′-tccttgattggtggaagctc-3′, reverse primer, 5′-gagggtcgacaaagggttg-3′, probe #92; *MCP-1/Ccl2*, forward primer, 5′-catccacgtgttggctca-3′, reverse primer, 5′-gatcatcttgctggtgaatgagt-3′, probe #62; *GAPDH*, forward primer, 5′-agcttgtcatcaacgggaag-3′, reverse primer, 5′-tttgatgttagtggggtctcg-3′, probe #9. The relative expression of each gene of interest was calculated from triplicate samples using the comparative threshold cycle number and normalized to the *GAPDH* internal control.

### Statistical evaluation

Data representing the mean ± standard deviation for the results of at least three independent experiments were compared by Student’s *t* test. Values were considered statistically significant if *p* was less than 0.05. The regression line between area ratio of ssDNA^+^ in the ONL and area ratio of fundus whitening was calculated. Statistical analysis was conducted using the commercial software package Excel (Microsoft, WA, USA) and Statcel3 (Add-in forms on Excel, H. Yanai, OMS, Tokyo, Japan).

## Supplementary information

Supplementary Information.

## Data Availability

The datasets generated during and/or analyzed during the current study are available from the corresponding author on reasonable request.
