# Supplementary material for: Microglia dynamics in retinitis pigmentosa model: formation of fundus whitening and autofluorescence as an indicator of activity of retinal degeneration
Source: Sci Rep. 2020 Sep 7;10:14700. doi: 10.1038/s41598-020-71626-2 (PMC7477572; doi:10.1038/s41598-020-71626-2)
Supplement: Supplementary file 1 — Supplementary Information. [file 41598_2020_71626_MOESM1_ESM.pdf]

Scientific Reports

Supplemental Information

Microglia dynamics in retinitis pigmentosa model; Formation of fundus whitening and autofluorescence as an Indicator of activity of retinal degeneration

Kenichi Makabe, Sunao Sugita, Michiko Mandai, Yoko Futatsugi, and Masayo Takahashi

Inventory of Supplemental Information

Supplemental Figure 1, fundus imaging and retinal pathology of wild type mice
Supplemental Figure 2, sequential fundus imaging in the same *rd10* mouse
Supplemental Figure 3, related to Figure 2B, 3B, 4B and 5B
Supplemental Figure 4, reactivation of retinal degeneration by light environment change
Supplemental Figure 5, time course of microglia activation in the retina of *rd10* mice
Supplemental Figure 6, expressions and localizations of MCP-1 in the retina of *rd10* mice.
Supplemental Figure 7, involvement of RPE cells in fundus autofluorescence

Supplemental Figure 1.

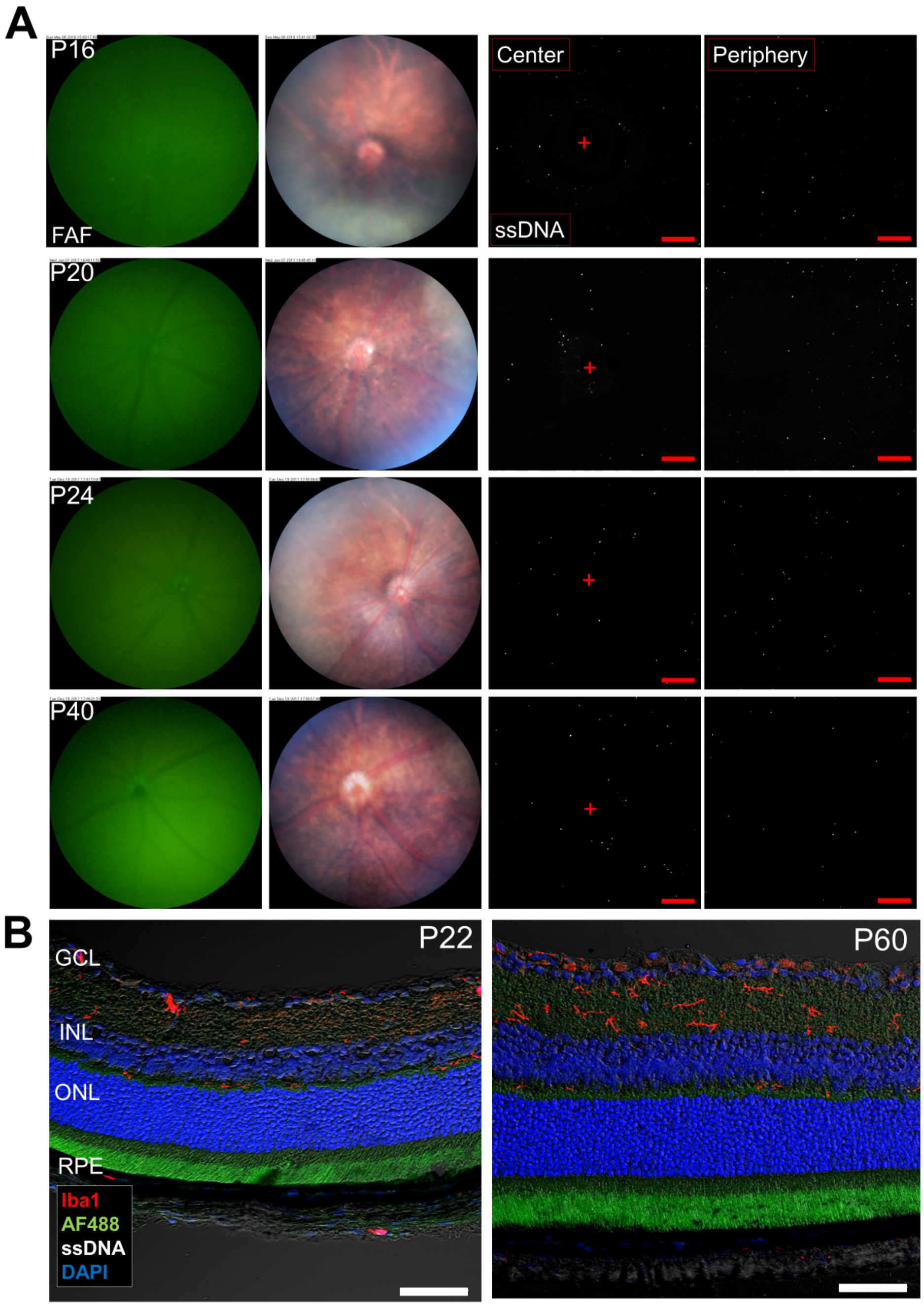

Fundus imaging and histopathological findings in wild type mice.

(A) Photographs show time course change in the retina of C57BL/6J mice from P16 to P40; *in vivo* fundus autofluorecence imaging with a retinal imaging microscope (left), color

fundus imaging (middle) and confocal images of ssDNA-labeled flat-mounted retina at the ONL level (right two rows; Scale bars, 100 μm, "+" shows the center of the optic nerve head). No fundus whitening and no fundus autofluorescent spots was observed during the time course. There were only a few $ssDNA^+$ cells during the time course. **(B)** Retinal sections of C57BL/6J mice on P22 and P60 labeled with Iba1, ssDNA and DAPI. There was no infiltration of microglia into the ONL. During the time course, the ONL was maintained without thinning. Scale bars, 50 μm.

Supplemental Figure 2.

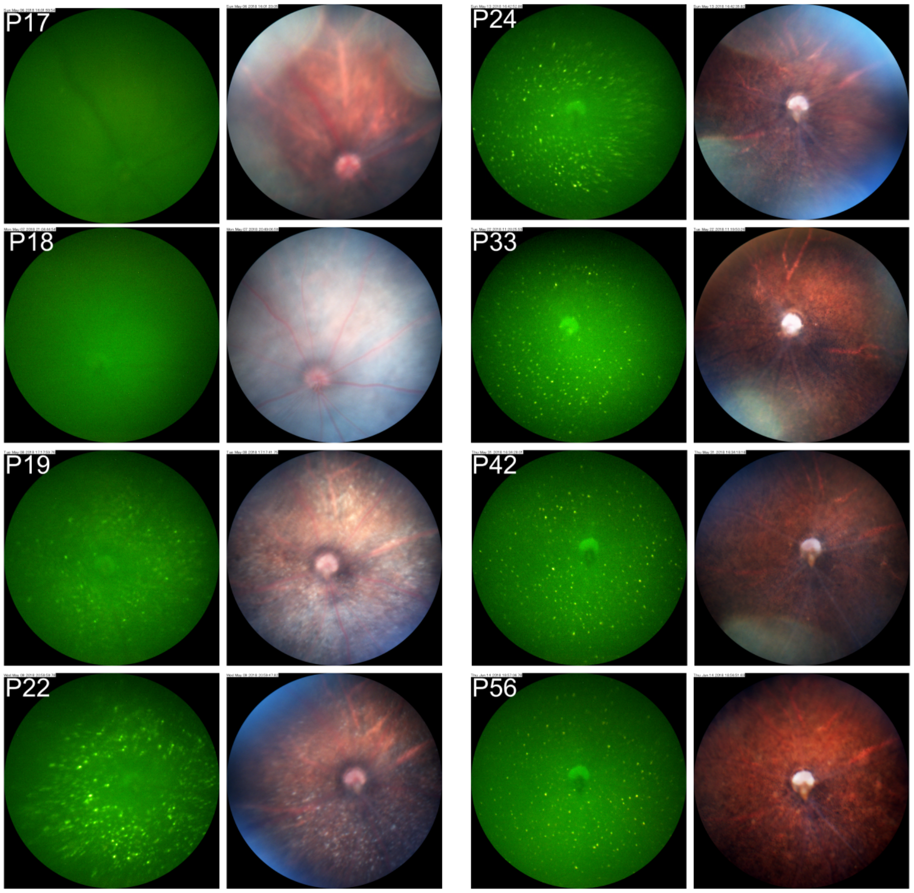

Time course change of fundus autofluorescence (FAF) spots and fundus whitening in *rd10* mouse.

Photomicrographs showing time course of FAF imaging (left) and color fundus imaging (right) in the same *rd10* mouse. At P18 the fundus became white, and the next day fluorescent spots appeared. In the color fundus photograph, white spots coincided with the fluorescent spots of FAF imaging. The number of fluorescent spots peaked on P22.

Supplemental Figure 3.

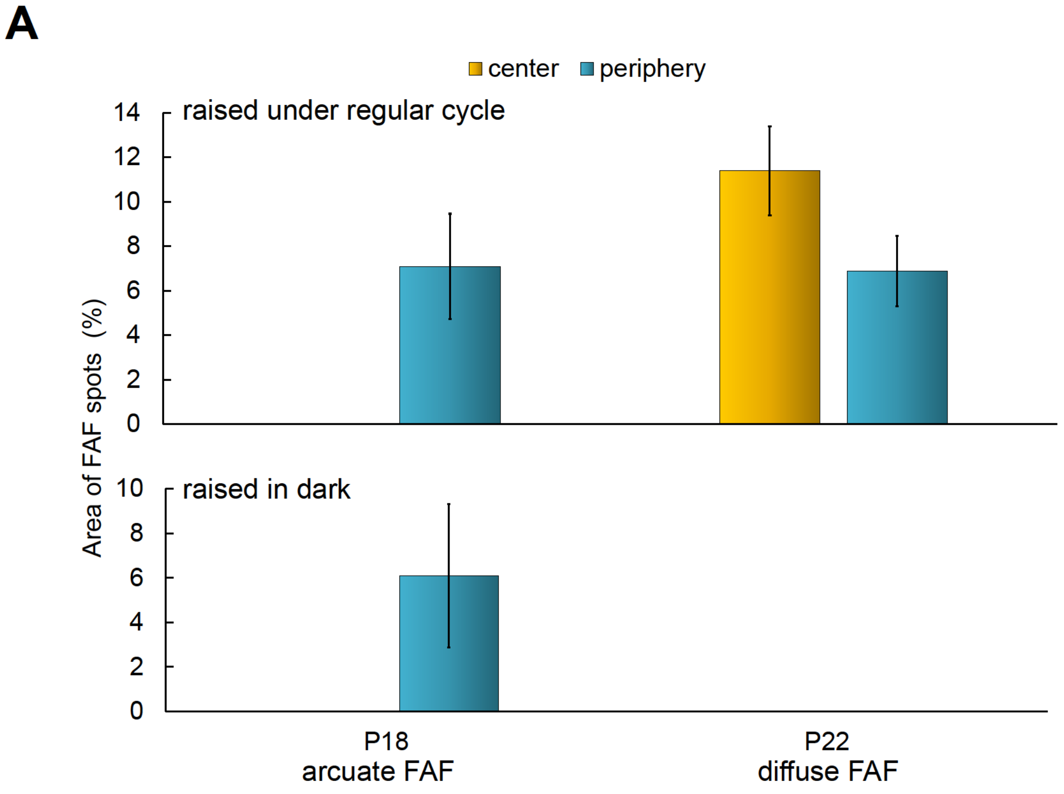

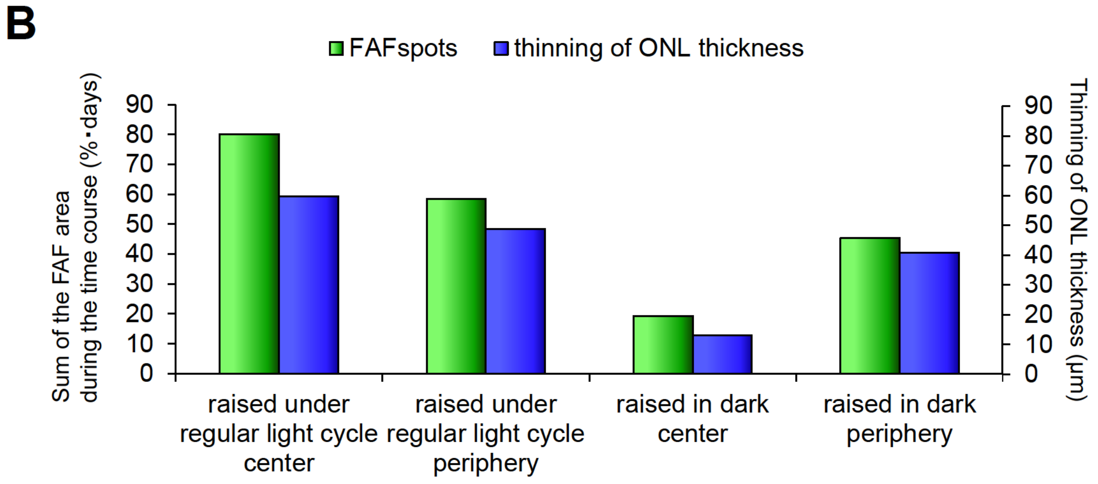

Two types of fundus autofluorescence spots and decrease of photoreceptors correlated to the FAF spots in *rd10* retina.

(A) Bar graphs show the area of arcuate FAF spots of *rd10* at P18 and diffuse FAF spots at P22 raised under different light environment. (n = 4, Error bars indicate mean ± SD). Arcuate FAF spots appeared exclusively in the periphery regardless of the light environment. Diffuse FAF spots appeared after arcuate FAF spots although they were suppressed if raised in dark. **(B)** Green bars show the sum of FAF spots during the time course (from P16 to P60) in either the center or the periphery under different light environment (Related to Fig. 2B, 3B, 4B and 5B). Blue bars show the thinning amount of the ONL from P16 to P60 (related to Fig. 2B, 3B, 4B and 5B). Both amounts showed a similar trend.

Supplemental Figure 4.

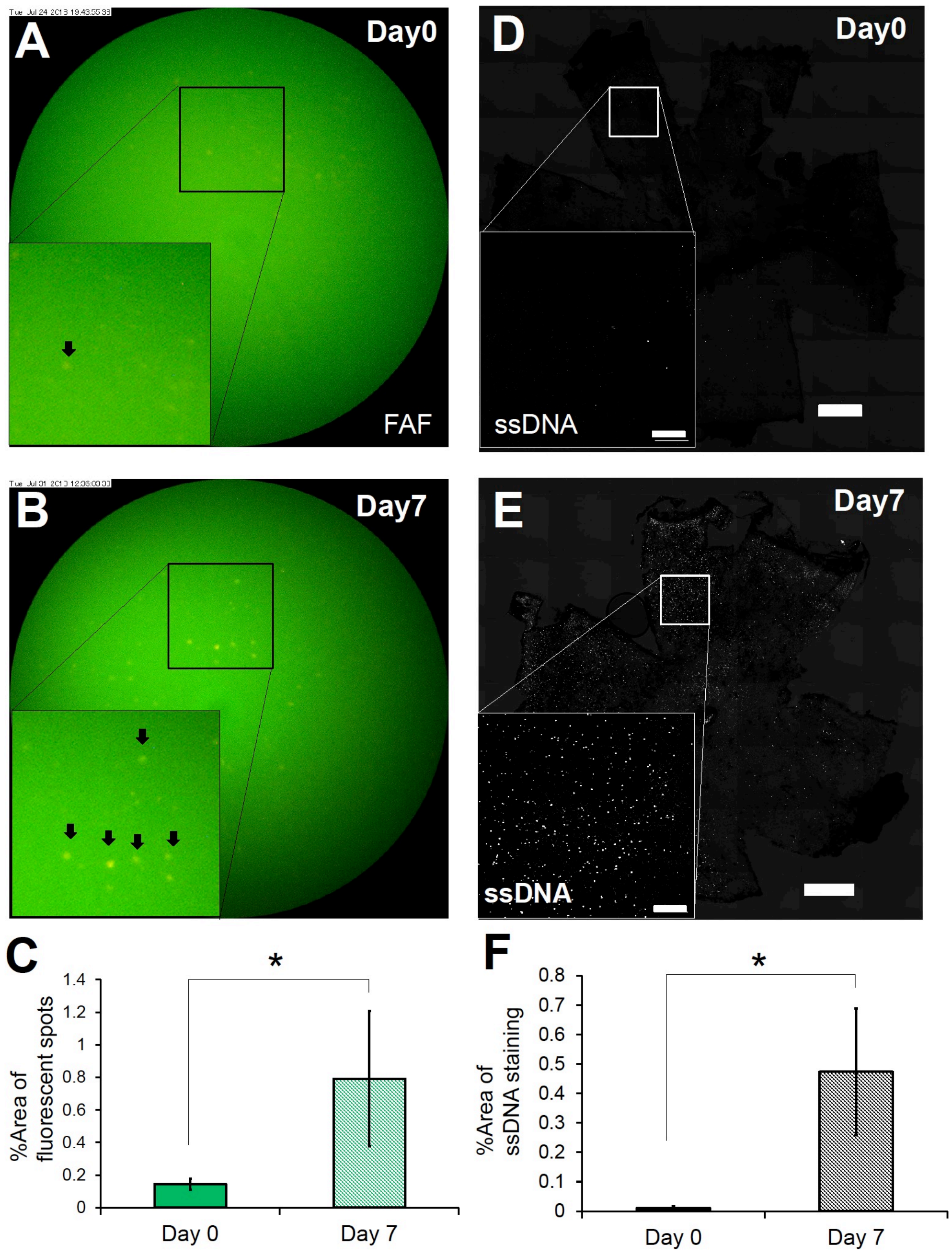

Reactivation of retinal degeneration and autofluorescent spots by light environment change.

In vivo FAF imaging (left) revealed the change in FAF spots of *rd10* mice by changing the light environment from dark to regular light cycle. **(A)** FAF spots of the dark raised *rd10* mice at postnatal 10 months. The spots were only slightly visible (Day 0). **(B)** Seven days after changing to regular light cycle (Day 7). FAF spots were more clearly observed. **(C)** The graphs show the comparison in the fluorescent spot area between Day 0 and Day 7. Significant increase in autofluorescent spots were observed (n = 4, Error bar indicate mean ± SD, *p < 0.05). %area, area density (%).

The right photographs show changes in ssDNA immunostaining of flat-mounted *rd10* retina with light environment change. Scale Bars, 500 µm. (Scale bars of magnified image, 100 µm). The *rd10* Day 0 shows only a few $ssDNA^+$ apoptotic cells **(D)**, while on Day 7 $ssDNA^+$ cells ware observed diffusely **(E)**. **(F)** The graphs show the comparison in the area of $ssDNA^+$ immunostaining between Day 0 and Day 7. Significant increase in $ssDNA^+$ area was observed (n = 4, Error bar indicate mean ± SD, *p < 0.05).

Supplemental Figure 5.

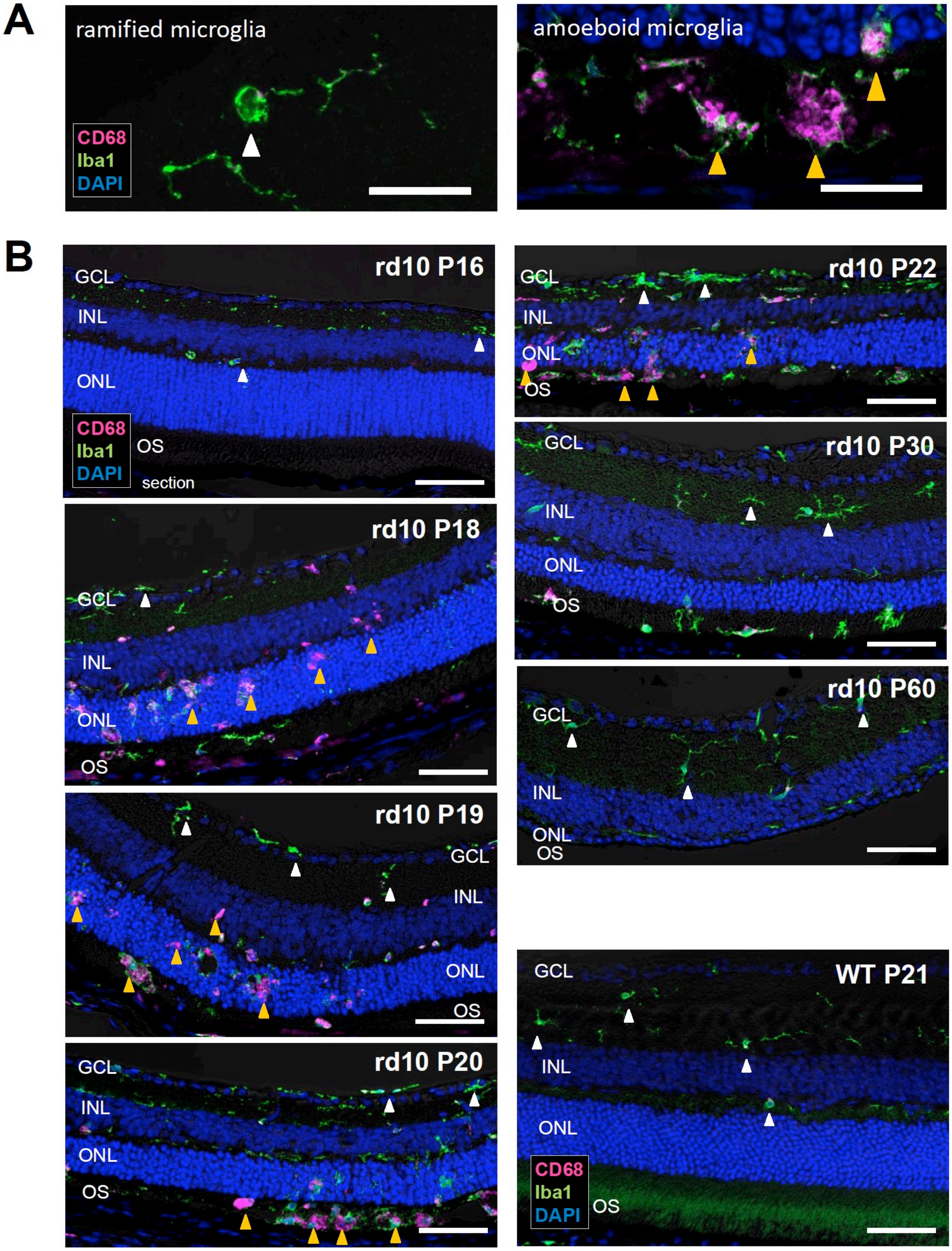

Activation of retinal microglia infiltrating into the layers of photoreceptors (ONL and OS) layer in *rd10* mice

(A) Magnified image of two types of microglia in the retina of *rd10* mice. CD68, a microglia activation marker, was most likely negative in ramified microglia. On the other hand, CD68 was positive in amoeboid microglia. Scale bars, 20 μm. **(B)** In the retina of *rd10*, microglia infiltrated into the photoreceptor layers (ONL and OS) during the period when degeneration of photoreceptors was active (P18 − P22). Microglia remaining in the inner layer of the retina were negative for CD68 (white arrowhead), while infiltrating microglia were amoeboid form and positive for CD68 (yellow arrowhead). In the retina of WT, all microglia were ramified type and CD68 negative (white arrowhead). Scale bars, 50 μm.

Supplemental Figure 6.

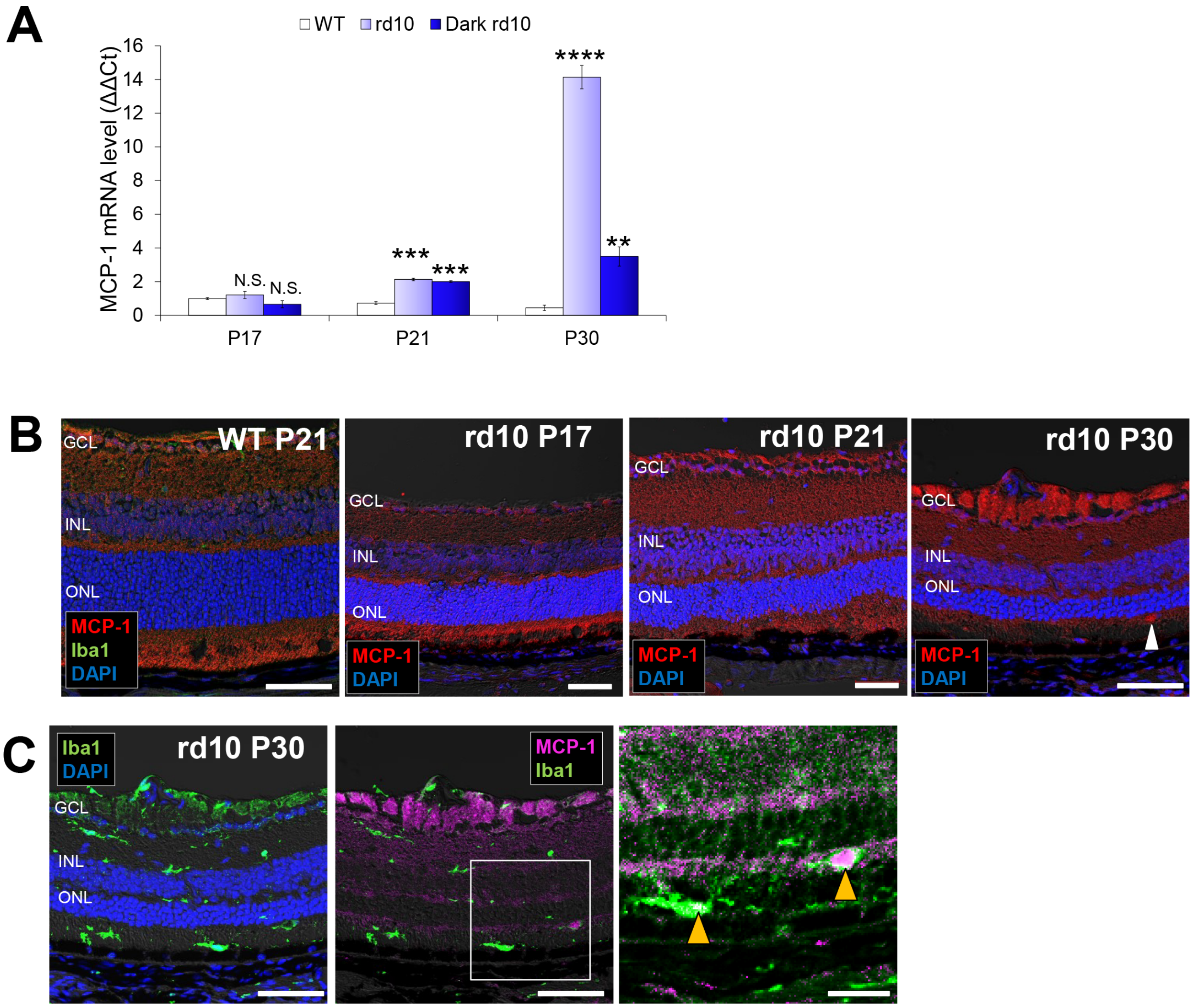

Expressions and localizations of MCP-1 in the retina of rd10 mice.

(A) Comparison of the mRNA expressions of MCP-1 in the retinas of WT C57BL/6J, rd10 raised under regular light cycle and rd10 raised in dark, by qRT-PCR. Results indicate the relative expression (ΔΔCt: WT P17 = 1). The graphs show mean and SD of three experiments (*$p < 0.05$, **$p < 0.005$, ***$p < 0.0005$, ****$p < 0.00005$ compared to WT). N.S., not significant. **(B)** Retinas of WT and rd10 labeled with MCP-1. Clear staining of MCP-1 was not observed in the retina of WT and rd10 on P17 and P21. On P30, some cells in the outer retina of rd10 (white arrowhead) were stained with MCP-1. Scale bars, 50 μm.

(C) Retina of *rd10* at P30 labeled with Iba1 and DAPI (left), and with MCP-1 and ssDNA (middle). Magnified image of the outer retina within the white square on the middle picture (right). $Iba1^{+}$ cells scattered in the whole retina, and the localization of some $Iba1^{+}$ cells in the outer retina coincided with the staining of MCP-1 (yellow arrowheads). Scale bars, (left and middle) 50 µm, (right) 20 µm.

Supplemental Figure 7.

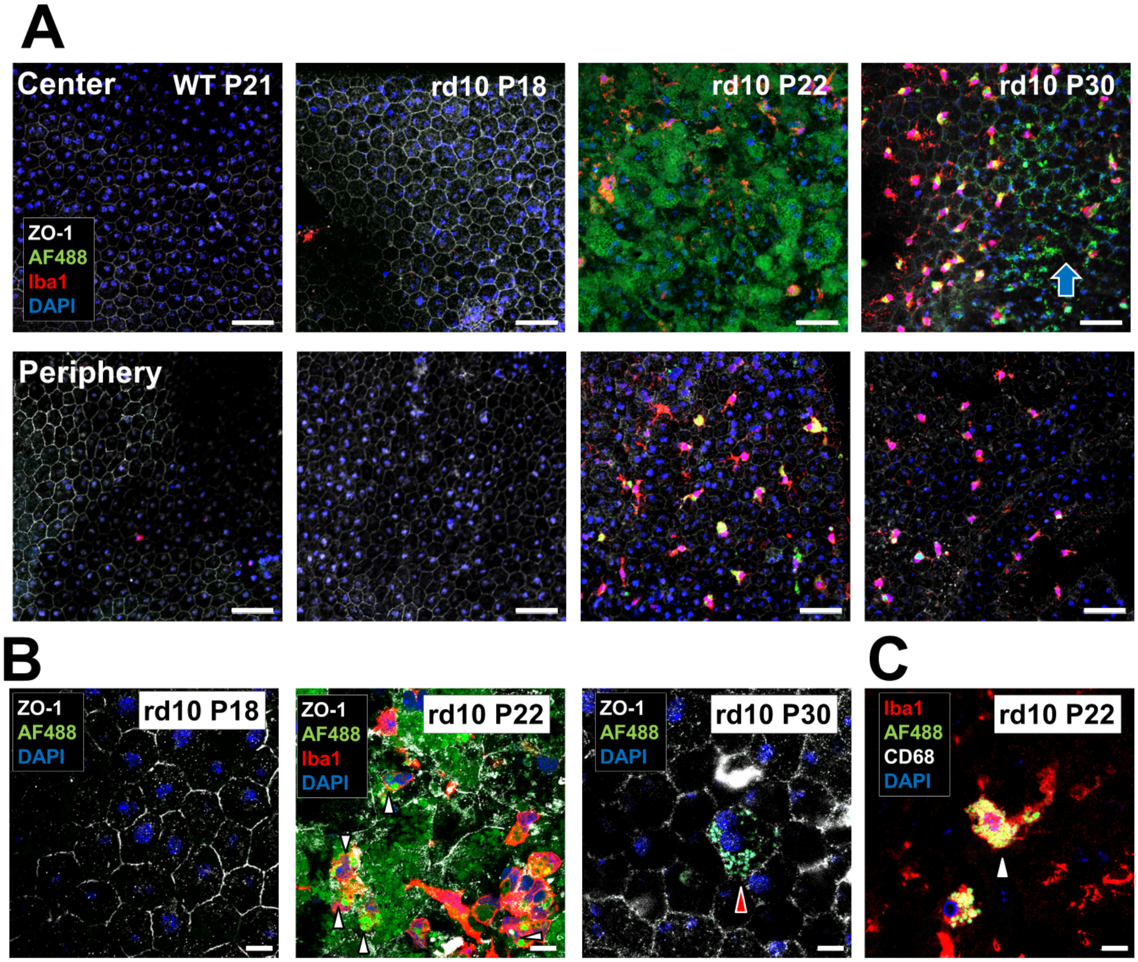

Involvement of retinal pigment epithelium (RPE) cells in fundus autofluorescence.

(A) RPE cell layers of WT C57BL/6J (P21) and *rd10* (P18 to P30) labeled with ZO-1, Iba1 and DAPI. There was no autofluorescent RPE cell in the retina of C57BL/6J and *rd10* at P18. In the central part of *rd10* at P22, RPE expressing ZO-1 became a large irregular shape, and many RPE cells emitted dim autofluorescence (enlarged image in **B**). In the peripheral part, exclusively Iba1$^+$ cells formed autofluorescent spots. In the retina of *rd10* P30, many fluorescent spots coincided with Iba1$^+$ cells, but there were some RPE cells that emitted autofluorescence (arrow; enlarged image in **B**). There was no RPE cell that emitted autofluorescence in the periphery. Scale bars, 50 μm. **(B)** Photomicrographs showing the magnified images of the flat-mounted RPE cell layer of *rd10* mice labeled with ZO-1, Iba-1

and DAPI. ZO-1$^+$ RPE cells of *rd10* at P18 formed a hexagonal sheet and did not emit autofluorescence. RPE cells of *rd10* at P22 became large and emitted low level of autofluorescence. However there were also some autofluorescent spots with high intensities (white arrowheads) which were comparable to those in Iba1$^+$ cells. Some RPE cells of *rd10* at P30 had autofluorescent granules (red arrowhead). Scale bar, 10 μm. **(C)** Photomicrographs showing the magnified image of the autofluorescent microglia on the flat-mounted RPE cell layer of *rd10* mice labeled with Iba1, CD68 and DAPI. The autofluorescent spots of microglia coincided with autofluorescent granules within the CD68$^+$ phagosome. The appearance of autofluorescent granules in microglia were similar to those in RPE cells (**B**, red arrowhead). Scale bars, 10 μm.
